# Oromotor functional networks in the marmoset monkey

**DOI:** 10.1162/IMAG.a.1419

**Published:** 2026-10-26

**Authors:** Azadeh Jafari, Audrey Dureux, Alessandro Zanini, Amr F.K. Eed, Michael R. Corigliano, Meng-Kuan Lin, David J. Schaeffer, Ravi S. Menon, Stefan Everling

**Affiliations:** Centre for Functional and Metabolic Mapping, Robarts Research Institute, University of Western Ontario, London, ON, Canada; Department of Neurobiology, University of Pittsburgh, Pittsburgh, PA, United States; Department of Physiology and Pharmacology, University of Western Ontario, London, ON, Canada

**Keywords:** awake marmoset, whole-brain fMRI, oromotor control, conspecific vocalizations, multimodal data integration, deep learning, markerless pose estimation

## Abstract

Primate communication relies on the precise coordination of the orofacial complex. While New World monkeys are growing models for vocal communication research, the neural basis underlying their oromotor control remains underexplored. Here, we used ultra-high-field fMRI in awake common marmosets (*Callithrix jacchus*) to map the whole-brain neural networks engaged during spontaneous visible oromotor functions—including jaw and lip movements, tongue protrusion, swallowing, and vocalization—distinct from those recruited during the auditory processing of conspecific calls. The resulting oromotor maps reveal a widespread control substrate extending beyond primary sensorimotor cortices, encompassing dorsal prefrontal (areas 8aD, 8b), mid-cingulate, posterior parietal areas (PF, PFG, and PE), and auditory regions. The parietal engagement points to a role for sensorimotor integration in marmoset oromotor control. Subcortically, this network engages the thalamus, basal ganglia, and cerebellum, with additional activation in the medullary reticular formation and periaqueductal gray, brainstem regions implicated in rhythmic oromotor control. A predominantly dorsal pattern of activation, with limited ventral premotor engagement, defines this network and provides a foundational framework for future studies of vocal–motor control in this species and a comparative reference for the rhythmic oromotor substrates from which speech is hypothesized to have evolved.

## 1 Introduction

The orofacial complex serves as a key anatomical interface for respiratory, sensory, feeding, and communicative functions (Sherwood et al., 2005). Across primates, ecological and social conditions have driven marked diversification in the structure and kinematics of this region, giving rise to species-specific orofacial behaviors, including facial expression, mastication (i.e., chewing), swallowing, jaw and lip movements, vocalization, and speech production, all of which are vital to their daily life (Avivi-Arber et al., 2011; Sherwood et al., 2005). Coordinated interactions among the jaw, lips, and other articulators give rise to oromotor functions, which control the shape and resonance properties of the supralaryngeal vocal tract (SVT) (Hauser & Ybarra, 1994; Lindblom & Sundberg, 1971). The execution of these movements relies on an interconnected neural network that integrates motor commands with sensory inputs and somatosensory feedback (Moore et al., 2014; Vázquez et al., 2025).

Importantly, human speech shares overlapping neural substrates, muscular activation patterns, and kinematic features with other oromotor functions often referred to as speech-like or nonspeech behaviors (Kent, 2015). Consistent with this view, human fMRI findings have shown that producing a single vowel and performing mouth opening recruit largely overlapping motor networks (Sörös et al., 2006). Further neuroimaging evidence also demonstrates that movements of the human lips, jaw, tongue, or larynx draw on a common cortical and subcortical network (Grabski et al., 2012), indicating that speech production and basic non-vocal oromotor behaviors may rely on a shared neural circuitry.

This neural overlap has long inspired evolutionary hypotheses suggesting that speech rhythms derive from the cyclic orofacial movements of ancestral primates. In this view, movements used during ingestion, such as mastication, were later adapted into the open–close articulatory patterns underlying syllable production (Ghazanfar & Takahashi, 2014; MacNeilage, 1998; Morrill et al., 2012). This transition may have involved extensive reorganization of the SVT to allow for rapid articulation, possibly accompanied by adaptations within the brainstem cranial motor nuclei (Sherwood et al., 2005) that provide motor innervations to the SVT (Jürgens, 2002).

Supporting this evolutionary framework, many nonhuman primates (NHPs) exhibit non-vocal oromotor behaviors (e.g., lip smacking, characterized by rhythmic jaw and lip movements) that share temporal, motor, and developmental features with the basic patterns of human speech (Bergman, 2013; Ghazanfar & Takahashi, 2014; MacNeilage, 1998; Morrill et al., 2012). These commonalities suggest that human speech may have evolved from conserved oromotor systems (MacNeilage, 1998; Risueno-Segovia & Hage, 2020). NHPs, therefore, provide a valuable comparative model for investigating the neural pathways that control vocal and non-vocal oromotor functions. A substantial body of work examining these pathways as potential precursors of human speech has translational relevance for both speech production and oral motor disorders (Ghazanfar & Takahashi, 2014; Kent, 2015).

The common marmoset (*Callithrix jacchus*) displays rich oromotor behaviors (Correia-Caeiro et al., 2022; Eliades & Miller, 2017; Stevenson & Poole, 1976) and shares a comparable motor cortical and subcortical organization with other primates (Matsuzaki & Ebina, 2020). Having diverged from Old World primates around 35 million years ago (Miller et al., 2016; Schrago & Russo, 2003), it provides an independent comparative model for examining whether oromotor circuits identified in other primates are broadly conserved. However, the whole-brain organization of oromotor control in this species remains poorly characterized.

At the cortical level, both lateral and medial frontal areas send direct corticobulbar projections to the facial motor nucleus in macaques (Morecraft et al., 2001), and interact during facial movements as part of an integrated face sensorimotor circuit (Vázquez et al., 2025). Recent frameworks propose dorsomedial and ventrolateral frontal components for vocal control in primates, with ventrolateral regions—including ventral premotor (PMv) and ventrolateral prefrontal cortices (vlPFC)—supporting conditional selection of vocal/non-vocal oromotor responses and dorsomedial regions—including mid-cingulate cortex and the supplementary motor area (SMA)—contributing to outcome monitoring and behavioral adjustment (Loh et al., 2017). Consistent with these findings, multiple premotor areas and primary motor cortex (M1) project to the brainstem vocal pattern generator in both macaques and marmosets, with comparatively larger descending outputs from area 6 V and SMA in marmosets (Cerkevich et al., 2022). Marmoset premotor neurons are also modulated during self-initiated vocalizations, with some dedicated to vocal production and others shared with non-vocal oromotor movements (Roy et al., 2016).

Complementing these cortical pathways, recent work in Old and New World monkeys has outlined a limbic–midbrain–brainstem pathway for vocal production and oromotor control (Hage et al., 2025; Moore et al., 2014; Sawczuk & Mosier, 2001; Sherwood et al., 2005). Electrical stimulation and tract-tracing studies showed that the anterior cingulate cortex (ACC; areas 24/32) can elicit vocalizations and projects to the periaqueductal gray (PAG), which in turn influences premotor neurons in the pontomedullary reticular formation and nucleus ambiguus that drive laryngeal and orofacial motoneurons (Hage, 2009; Jürgens, 2009; Jürgens & Pratt, 1979b; Jürgens et al., 1967; Larson & Kistler, 1986; Müller-Preuss & Jürgens, 1976; Robinson, 1967; W. K. Smith, 1945). Building on this foundational work, hierarchical models propose that ACC and prefrontal cortex form a higher-order vocal motor network that exerts volitional control over this brainstem pattern-generating circuitry (Hage & Nieder, 2016; Jürgens, 2009). Together, these frameworks suggest that oromotor actions in primates should recruit not only primary sensorimotor cortices but also cingulate, prefrontal, cerebellar, and midbrain/brainstem centers.

In a recent study examining call-specific activations in awake head-fixed marmosets (Jafari et al., 2025), we observed that the animals occasionally produced spontaneous orofacial movements. These consisted of a range of behaviors, including facial expressions, jaw and lip movement (without vocalization), tongue protrusion and retraction, swallowing, and vocalization. Here, we focused specifically on the neural correlates of the visible oromotor movements (defined here as jaw/lip movements, tongue in–out, swallowing, and vocal production; see examples for two monkeys in Fig. 1B). While animals passively listened to conspecific calls, they also produced spontaneous oromotor movements, whose timing was quantified using markerless video tracking (DeepLabCut) to obtain precise movement onsets. We exploited ultra-high-field (9.4T) fMRI in fully awake common marmosets and used these timing estimates to (i) map, at the whole-brain level, the network engaged by spontaneous oromotor movements, (ii) examine whether key nodes of the classical vocal pathway (ACC, PAG, medullary reticular formation) are recruited during these oromotor behaviors, and (iii) compare this oromotor network with that engaged during passive listening to conspecific calls, building on the functional organization of passive auditory processing established in our previous fMRI studies (Dureux et al., 2024; Jafari et al., 2023, 2025).

## 2 Materials and Methods

### 2.1 Common marmoset subjects

All experimental procedures followed the guidelines of the Canadian Council of Animal Care and were approved by the Animal Care Committee at the University of Western Ontario. In this study, 13 awake marmosets (*Callithrix jacchus*; 6 females; age 42–74 months; weight 380–464 g) were scanned. The marmosets were housed in pairs in a colony at the University of Western Ontario, with a 12-hour light/dark cycle, 24–26°C temperature, and 40–70% humidity.

### 2.2 Surgical procedure

To eliminate head movement during imaging sessions, a custom-machined PEEK (polyetheretherketone) head-fixation post was surgically implanted on the skull of each animal under aseptic conditions. During surgery, animals were sedated and then maintained under gas anesthesia (isoflurane). Following a 3-week recovery period, the monkeys underwent gradual habituation to the head-fixation system and MRI noise environment using a mock scanner setup. Detailed information on the surgical procedures and post-operative training protocols can be found in our methodological publication (Zanini et al., 2023).

### 2.3 Imaging setup

fMRI scans of awake marmosets were carried out at the Centre for Functional and Metabolic Mapping (CFMM) at the University of Western Ontario. Scanning was performed on an ultra-high-field magnetic resonance system 9.4T/31 cm horizontal bore magnet (Varian/Agilent) with a Bruker BioSpec Avance III console running Paravision 7 software package (Bruker BioSpin Corp). We used a custom-built gradient coil with a 15-cm inner diameter (maximum gradient strength 400 mT/m) and an eight-channel receive RF coil tailored for marmoset heads (Gilbert et al., 2023).

Awake marmosets were placed in a custom 3D-printed plastic chair inside the magnet, where an adjustable collar and tail plate maintained the animals in a comfortable sphinx position (Schaeffer et al., 2022; Zanini et al., 2023). Auditory stimuli were passively presented via MRI-compatible earphones (S14, Sensimetrics, Gloucester, MA) inserted into both ear canals and secured with reusable silicone earplugs and self-adhesive bandages to attenuate scanner noise. The RF coil housing was positioned around the animal’s head. Throughout the scan sessions, each marmoset was continuously monitored via an MRI-compatible camera with integrated infrared LED illumination (12M-I, MRC Systems GmbH) placed ~12 cm in front of the animal’s face. The camera feed was connected to a Raspberry Pi, and a custom Python script (using the OpenCV library; Bradski, 2000; Culjak et al., 2012) was used for frame-by-frame video recording. A trigger (transistor-transistor logic, TTL) pulse from the scanner synchronized the start of imaging, auditory stimulus presentation, and video capture. The camera sampled at 60 Hz at the sensor, and frames were saved through the Raspberry Pi pipeline at an effective rate of 20 Hz, which was the rate used for all subsequent video analysis. In a subset of 11 pilot runs, we also used an MRI-compatible fiber-optic microphone (Optoacoustics, OptiSLM series – Optimic™ 1155) to record audio inside the scanner. The microphone was positioned ~4 cm in front of the subject’s mouth and connected to a sound level meter and a laptop running Audacity (v3.2.5) (Audacity, 2017). The audio recording onset was synchronized with the start of both the imaging and video recording, and the audio was saved in waveform and Audacity project formats. Unfortunately, due to technical issues with the microphone setup, continuous audio recording could not be implemented during the acquisition of the main dataset. Cardiac and respiratory signals were not recorded during scanning in this study.

### 2.4 Functional and anatomical data parameters

To reduce the influence of scanner noise on the auditory stimuli, we implemented a bunched acquisition technique (as detailed in Jafari et al., 2023) for all task-based functional imaging. This involved inserting a silent gap (*TS*) after each volume acquisition time (*TA*), yielding an effective repetition time *TR* = *TA* + *TS*. In this study, we used gradient-echo-based single-shot echo-planar imaging (EPI) sequences for functional scans with the following parameters: *TA* = 1.5 seconds, *TS* = 1.5 seconds (*TR* = 3 seconds), echo time *TE* = 15 ms, flip angle = 40°, field of view = 64 × 48 mm, matrix = 96 × 128, voxel size = 0.5 mm isotropic, 42 axial slices, and bandwidth = 400 kHz. We employed GRAPPA (R = 2, phase-encode direction L-R) to shorten echo trains. In each fMRI session, an additional set of EPI volumes was acquired with reverse phase encoding (R-L) to allow for spatial distortion correction during preprocessing analysis. A T2-weighted anatomical image was also obtained for each animal (in a separate session) to aid anatomical alignment with the following parameters: *TR* = 7,000 ms, *TE* = 52 ms, field of view = 51.2 × 51.2 mm, voxel size = 0.133 × 0.133 × 0.5 mm, 45 axial slices, bandwidth = 50 kHz, GRAPPA R = 2.

### 2.5 Auditory stimuli and design

Details of the conspecific call stimuli and task design were previously described in Jafari et al. (2025). Briefly, the auditory stimuli consisted of 600-ms recordings of various marmoset call types from our colony (phee, twitter, trill, chatter), normalized for amplitude. Each run lasted 900 seconds, during which 300 functional volumes were acquired (*TR* = 3 seconds). Calls were played in random order during the silent periods between volume acquisitions (*TS*), with inter-stimulus intervals pseudorandomly chosen from 3, 6, 9, or 12 seconds. A custom Python script controlled sound presentation onset via a TTL pulse from the scanner, which also triggered video recording via the Raspberry Pi (through OpenCV (Bradski, 2000; Culjak et al., 2012)). Each monkey underwent 2–3 runs per session; in total, 119 runs were collected from 13 monkeys. After excluding 29 runs that lacked detectable oromotor activity (e.g., the monkey remained still or fell asleep) or had poor video quality, 90 valid runs from 11 monkeys were retained for examining both the oromotor activities (spontaneous visible articulator movements) and the conspecific auditory conditions (listening to marmoset calls).

### 2.6 Analyzing videos using DeepLabCut

To quantify the marmosets’ oromotor movements, we used DeepLabCut (DLC) (Mathis et al., 2018), a deep-learning tool for markerless pose estimation. DLC applies transfer learning, in which a pretrained deep neural network is fine-tuned on a small set of manually labeled frames, allowing accurate landmark tracking without physical markers and with limited training data (Mathis et al., 2018). Using DLC’s graphical user interface (Nath et al., 2019), we analyzed the recorded videos to track facial landmarks. We focused on six points on the face (see Fig. 1C): the upper lip midpoint (“U” in purple), lower lip midpoint (“L” in blue), left mouth corner (“LC” in cyan), right mouth corner (“RC” in green), and two reference points at the inner tips of the left (“Ref1” in orange) and right (“Ref2” in red) eyes. We first trained the network on example frames. For training the DLC network, we drew from the pilot videos not included in the main dataset (with audio)—selecting 20 frames per video from 11 videos (220 frames in total)—and manually labeled the 6 points of interest on each frame (Fig. 1C, left column). The labeled frames were then split into training and testing sets to train the DLC neural network (ResNet-50). Network performance was evaluated by the Mean Absolute Error between user’s labels and DLC’s predicted positions for each point. We enabled DLC’s plotting of training vs. test predictions for visual verification (Fig. 1C, right column), where plus signs mark user labels and dots mark DLC predictions (colored by body part, only plotted if confident predictions > p-cutoff) (Nath et al., 2019).

After confirming network accuracy, we applied the trained DLC network to the actual video dataset (90 runs). This yielded time series of 2D coordinates for each landmark in each frame. We found that the y-coordinate of the lower lip midpoint (L_Y_) and the x-coordinates of the mouth corners (LC_X_ and RC_X_) showed the largest frame-to-frame deviations—corresponding closely to oromotor movements in the videos. In contrast, the upper lip midpoint and reference eye points showed much smaller frame-to-frame variation than the other landmarks (see Fig. 1D). Based on these observations, we focused subsequent analyses for detecting oromotor movements on L_Y_ and RC_X_ (since left/right was largely similar). As shown in Figure 1E, L_Y_ and RC_X_ varied continuously across frames, but the magnitude of these positional changes did not show a clear separation that could distinguish different movement types. Because continuous audio was not available for the main dataset, and because marmosets can vocalize with the mouth nearly closed, vocal events could not be identified from video alone. Moreover, the audio-recorded sample (from 11 pilot runs) was too small to train a classifier reliably on mouth kinematics. We, therefore, treated all detected oromotor events as a single combined behavioral class in the fMRI analysis (i.e., we did not sub-categorize movements by type).

### 2.7 Identifying time points (regressors) of oromotor movements

Using the DLC-derived coordinate data, we next identified the time points (in the fMRI time series) where oromotor movements occurred. We extracted the frame-by-frame series of the lower lip’s y-position and the right mouth corner’s x-position for every run and monkey. A custom Python script was used to compute the mean and standard deviation (SD) of these coordinates for each run, and the traces were normalized to these run-wise values. Because the SD is computed from the traces, the same multiple of SD does not correspond to the same displacement in every run, and runs with larger articulator movements would have correspondingly larger SDs. The traces for L_Y_ and RC_X_ also differ, since not every movement displaces both landmarks to the same extent. The detection threshold was, therefore, not fixed across the dataset but determined from the data of each trace, and took values between 2 and 6 SD.

For each trace, we first detected the candidate frames at 2 SD and inspected them against the video, removing those frames that did not correspond to a visible articulator movement. Among those that remained, we identified the one with the smallest deviation from the run mean and computed its deviation in SD units. This value was used as the detection threshold for that trace. Within a session, the animal and its head and the camera remained in a fixed position across all runs, so the acquired threshold in one run could be ideally applied to the remaining runs as the start threshold and recomputed following the same procedure when it performed poorly.

Event detection was performed on both L_Y_ and RC_X_, and the two candidate lists were then pooled, so that a frame entered the movement list if either trace crossed its threshold; this was necessary because not all movements displace both landmarks equally, as when a vertical jaw opening moves the lower lip substantially while the mouth corner shifts little. Frame indices (60 frames per *TR*) were then converted to time (seconds) relative to the scan start as t = (frame index)/20 Hz, yielding a list of putative oromotor movement onset times for each run.

If multiple movement frames were separated by less than 1 second, we merged them and took only the first time point as the onset of a single movement event. This window was chosen because individual oromotor movements typically lasted on the order of a few hundred milliseconds to ~1 second, so that clustered frames from one sustained movement counted as a single event in the fMRI analysis. It also combined detections arising from the same movement on the two traces, since a given movement does not necessarily cross threshold on L_Y_ and RC_X_ at exactly the same frame. Because the number of movement events per run was relatively limited, we manually reviewed the complete video of each run against the detected list to ensure the identified events were true oromotor movements (and not false positives like brief camera jiggles). Any spurious detections were removed, genuine movements that had not been captured by the threshold were added, and detections that the merging step had combined although the video showed two distinct movements were separated, before finalizing the event timing files. The final event list used to construct the regressors was, therefore, established by visual verification against the full video. Across these 90 runs, a total of 2,033 oromotor events were detected, with a median of 20 events per run. Oromotor event counts varied across animals, ranging from 48 to 399 total events per monkey (median 187). Of these events, 92 involved a visible discomfort or alarm expression, and the remainder consisted of neutral, non-distress behaviors, such as spontaneous jaw and lip movement, tongue in–out, and swallowing.

### 2.8 Preprocessing of fMRI data

Preprocessing of the MRI data was carried out using AFNI (Cox, 1996) and FSL (S. M. Smith et al., 2004). First, each monkey’s T2-weighted anatomical image was reoriented to a standard orientation. A manually drawn brain mask (created with FSLeyes (S. M. Smith et al., 2004)) was applied to the anatomical image to remove non-brain signal. The masked anatomy was then nonlinearly registered to the 3D NIH Marmoset Brain Atlas (NIH-MBA) (Liu et al., 2018) using Advanced Normalization Tools (ANTS; Avants et al., 2009). Functional EPI data were converted from DICOM to NIfTI format (using *dcm2niix*), reoriented (FSL; *fslswapdim*) to match the anatomical orientation, and then corrected for spatial distortion (FSL; *topup /applytopup*).

We addressed motion and slice timing as follows: we first removed large spikes in each time series (AFNI; *3dToutcount* to identify outliers and *3dDespike* to attenuate them), then applied slice-timing correction (AFNI; *3dTshift*). All volumes of each run were realigned to a reference volume (the middle volume of that run) for motion correction (AFNI; *3dvolreg*). We then convolved each volume with a 3D Gaussian kernel (full-width half-maximum = 1.5 mm) for spatial smoothing (AFNI; *3dmerge*). Finally, we applied a temporal band-pass filter (0.01–0.1 Hz) to reduce low-frequency drift and high-frequency noise (AFNI; *3dBandpass*). A mean functional image was generated for each run and aligned to the T2-weighted anatomical image using FSL’s linear registration (*FLIRT*, 6 degrees of freedom). The transformation matrices from these alignments and the ANTS anatomical-to-atlas warp were saved for later application to the statistical maps.

### 2.9 Functional maps

We generated functional activation maps using a General Linear Model approach. For each run, we included separate task regressors for the oromotor movements and for the conspecific auditory events, with each event modeled as an impulse at its onset and convolved with a canonical hemodynamic response function (HRF; AFNI *GAM(4,0.7)*, time-to-peak ≈2.8 seconds). As mentioned in Section 2.7, oromotor events were typically brief—on the order of a few hundred milliseconds to approximately 1 second—which is short relative to the HRF time-to-peak; impulse modeling is, therefore, appropriate at this temporal scale. Because all events were modeled identically as impulses, this approximation is expected to have a minor effect on the estimated BOLD responses. Event duration was, therefore, not used as an additional regressor in the present analyses. Third-order polynomial terms (to model baseline and drift) and six rigid-body motion parameters (from the motion correction step) were included as nuisance regressors. The design matrix for each run was assembled and fitted (AFNI; *3dDeconvolve*), yielding β-coefficients and associated *t*-statistics for each regressor. The resulting β-maps for each condition were then transformed to the template space by applying the previously computed affine and nonlinear transformations.

After registration to the NIH-MBA template (Liu et al., 2018), we obtained *t*-value maps for both the oromotor and conspecific auditory regressors for each run. For group-level comparisons, we first averaged the run-level maps within each subject. We then performed voxel-wise two-tailed paired *t*-tests across subjects (AFNI; *3dttest++*, with each subject’s oromotor vs. auditory contrast), yielding group *Z*-statistic maps for (i) *oromotor > baseline*, (ii) *conspecific auditory > baseline,* and (iii) *oromotor > auditory* (and the reverse). To visualize results, the group *Z*-maps were thresholded at a cluster-corrected significance level of *p* < 0.05 (family-wise error corrected), using cluster-size inference (AFNI; *3dClustSim*) with a voxel-wise threshold of *Z* > 2.3. These thresholded activation maps were overlaid onto marmoset brain templates for display. Surface projections of cortical activations were made on a 3D fiducial surface and a 2D flatmap using Connectome Workbench v1.5.0 (Marcus et al., 2011). For coronal and sagittal slice views, we used FSLeyes (S. M. Smith et al., 2004) with a high-resolution (100 µm) ex-vivo marmoset brain template as the underlay.

To identify the anatomical regions of activated clusters, we aligned our functional maps to the high-resolution atlas provided by the Marmoset Brain Connectivity Atlas project (an ex vivo MRI atlas co-registered to the NIH template) (Schaeffer et al., 2022) and used the Paxinos marmoset brain parcellation (Paxinos et al., 2012) for nomenclature. This allowed us to assign each activation cluster to a brain region, as reported in Section 3.

### 2.10 Separating camera motion from articulator movements

Because the marmosets were head fixed, any frame-to-frame displacement of the reference landmarks (Ref1, Ref2) primarily reflected motion of the camera—predominantly gradient-induced vibration during volume acquisition (*TA*)—which was more pronounced in our earlier sessions and largely resolved in subsequent acquisitions. A clear example is shown in Supplementary Figure S1, where a shift in the camera displaced the whole set of facial landmarks (Supplementary Fig. S1B), relative to a baseline frame in which the monkey was still, and the camera was stationary (Supplementary Fig. S1A). By contrast, during an actual mouth opening (without the effect of the camera motion), the reference landmarks remained close to their average position across the run (dashed lines), while the lower lip was substantially displaced (Supplementary Fig. S1C). Camera motion can, therefore, produce coordinate changes in the articulator traces that resemble those generated by genuine movements.

In the main analysis described in Section 2.7, movements that did not cross the threshold were identified during the full-video review and added to the event list, so the final event list did not require the traces to be fully corrected for camera motion. For the threshold-sensitivity analysis (see Section 2.11), however, we evaluated only the events detected by the fixed threshold itself and did not add back movements that it missed. Because camera vibration increases the run-wise SD, it raises the absolute displacement required to cross a given SD threshold and can cause smaller genuine movements to be missed. We, therefore, reduced the camera-motion contribution from the traces before performing this threshold-sensitivity analysis.

For each frame, we computed the centroid of Ref1 and Ref2 (the mean of their x- and y-coordinates) and then computed the run-wise mean of these per-frame centroids (the global centroid). For each frame, we subtracted the global centroid from that frame’s centroid, yielding the displacement of the camera (ΔX and ΔY) at that specific frame. To prevent transferring single-frame DLC localization errors from the reference points into the corrected articulator traces, we smoothed this displacement signal with a five-frame moving median (250 ms at 20 Hz). This window length is short enough to preserve camera shifts at the *TA*-locked timescale (1.5 second) while attenuating single-frame DLC prediction errors. The smoothed per-frame displacements were then subtracted from the lower lip y-coordinate and the right mouth corner x-coordinate of that frame to reduce the effect of camera jiggle on the articulator traces. This correction step was applied to the whole dataset (90 runs, 11 monkeys) for the threshold-sensitivity analyses reported below.

### 2.11 Sensitivity of the activation maps to the event-detection threshold

To assess whether the observed activation patterns were robust to the choice of event-detection threshold, we repeated the computation of the functional maps (*oromotor > baseline* contrast) using event sets defined by fixed thresholds. Unlike the main pipeline described in Section 2.7, in which the threshold was determined separately for each trace and run, a single fixed value of 2, 3.5, and 5 SD was applied to both L_Y_ and RC_X_ in this step, so that the threshold was the only variable between the three event sets. These three evenly spaced values (1.5 SD steps) span most of the 2–6 SD range used in the main analysis. We did not include 6 SD, as too few events were retained at that threshold for reliable estimation of the BOLD response.

At each threshold, candidate frames from both traces were acquired, pooled, converted to event onsets, and then merged as in the main pipeline, and each detected event was inspected against the video; detections that did not correspond to a visible articulator movement were removed. However, genuine movements that the threshold had failed to detect were not added back, since the purpose of this analysis is to characterize what the threshold alone recovers from the corrected traces.

Because a single fixed threshold was applied to every run and missed movements were not added back, these analyses were performed on the camera-motion-corrected traces obtained by the centroid correction step (see Section 2.10), so that a given multiple of the SD corresponded to comparable articulator displacement across runs.

Fewer events crossed the higher thresholds (3.5 SD and 5 SD), and some runs had no detected events at those levels. To keep the number of runs and animals the same across the 3 thresholds, we used 43 runs from 9 monkeys in which oromotor events were detected at all 3 thresholds, yielding 1,332 events at 2 SD, 870 at 3.5 SD, and 648 at 5 SD. All other preprocessing, GLM, subject-level averaging, and group-level statistical procedures were identical to those used in the main analysis.

### 2.12 Cerebellar cartography

To visualize activation patterns across the cerebellum in detail, we utilized the Marmoset Brain Connectome project’s cerebellar atlas (Schaeffer et al., 2022). This atlas integrates histological and MRI data to delineate cerebellar anatomy at high resolution. In brief, the cerebellum was manually labeled on 25-µm ex vivo MR images aligned with 20-µm Nissl-stained sections of the same brain. Using the contrast differences between molecular and granular layers, a nearly distortion-free flatmap of the cerebellar cortex was created by computationally unfolding along each folium (Schaeffer et al., 2022). We resampled our functional cerebellar contrast map (oromotor vs. conspecific auditory) to the cerebellar atlas space and then projected it onto the flatmap (using the 3D-to-2D coordinate transform provided by the atlas). This enabled a convenient visualization of cerebellar activity across lobules on a 2D plane (Fig. 6). Further details of this cartographic technique are available via the online repository of the cerebellar atlas (Schaeffer et al., 2022).

## 3 Results

Marmosets were presented with brief conspecific calls (common phee, twitter, trill, and chatter calls) at pseudorandomized inter-stimulus intervals of 3, 6, 9, or 12 seconds. These calls evoked significant increases in the blood-oxygen-level-dependent (BOLD) signal in auditory regions—including the auditory cortex, inferior colliculus (IC), and medial geniculate nucleus (MG)—as shown in Figures 2B and 3B. Detailed results of this auditory-stimulus condition have been published elsewhere (Jafari et al., 2025). Simultaneous video footage analysis (Fig. 1A, top) revealed that while marmosets remained still without any orofacial movement during most volumes (e.g., volumes 2, 3, 5, and 6 in the example), several types of orofacial movements occurred during some volumes (e.g., volumes 1, 4, and 7). Our video analysis indicated that these movements encompassed mainly facial expressions, jaw and lip movements (without vocalization), tongue protrusion (in–out), swallowing, and vocalization. For instance, volumes 1 and 7 in Figure 1A (top) exhibited clear sound pressure waveforms and spectrogram features consistent with vocal production (Fig. 1A, bottom). In contrast, the movement identified in volume 4 was a silent jaw movement without accompanying vocalization. We focused our fMRI analysis on the oromotor movements, defined as fMRI volumes containing visible articulator motions (see examples in Fig. 1B), and compared these volumes with (i) a baseline period without any movement or conspecific call stimulation and (ii) volumes containing only conspecific auditory task events.

**Fig. 1.**
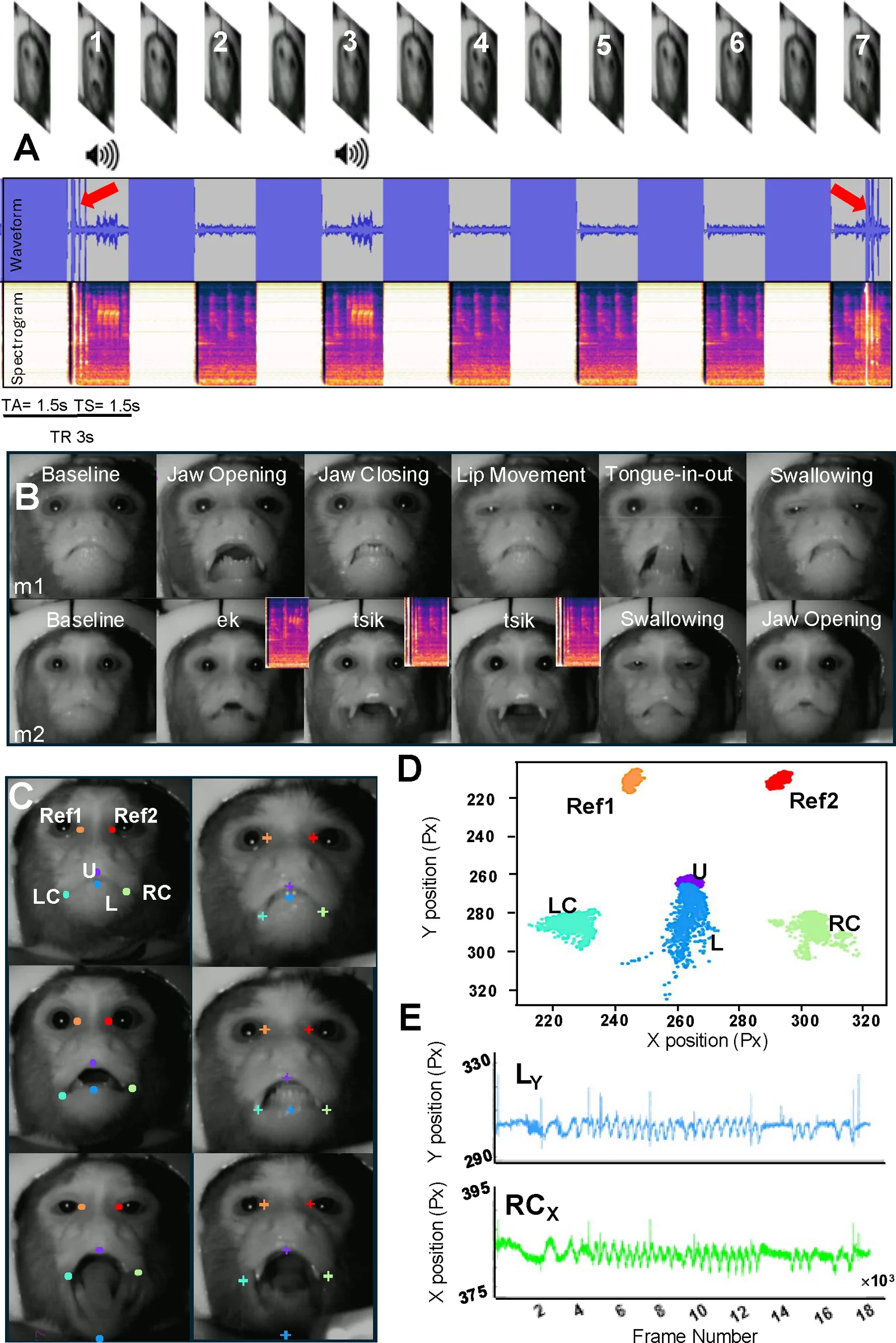
Oromotor movements in awake marmosets. (A) Examples of video frames (top), audio waveforms, and corresponding spectrograms (Audacity, 2017) (bottom) during seven successive fMRI volumes (21 seconds) in an awake marmoset. Red arrows mark spontaneous vocalizations in the waveform and spectrogram. Volumes 2, 3, 5, and 6 show no movement; volume 4 shows a silent jaw movement; volumes 1 and 7 show vocalizations (with spectral energy visible in the spectrogram). (B) Representative oromotor behaviors observed across two different monkeys (m1 and m2), drawn from the pilot runs in which simultaneous audio was recorded. The examples shown illustrate the range of behaviors detected and are not proportional to their frequency of occurrence: baseline (no movement), jaw opening, jaw closing, lip movement, tongue protrusion (tongue-in–out), swallowing, and vocalization. The vocalizations shown are *tsik* and *ek* calls, two marmoset call types associated with alert states, presented with an example spectrogram of each call. (C) DeepLabCut tracking of facial landmarks. Left panel: example video frame with manual annotations of key points (U = upper lip midpoint, purple; L = lower lip midpoint, blue; LC = left mouth corner, cyan; RC = right mouth corner, green; Ref1 = inner left eye corner, orange; Ref2 = inner right eye corner, red). Right panel: DLC output for a training frame, showing the network-predicted positions (colored small dots) versus user labels (colored +) for each landmark. Markers are only shown for high-confidence predictions (dots), as determined by a likelihood threshold. (D) Scatterplot of tracked positions (in pixels, Px) for one representative run. Coordinates are shown for U, L, LC, RC, and the reference eye points (Ref1, Ref2). Greater variability is seen in the y coordinates of L and x coordinates of LC and RC. (E) Landmark time series used for the event detection. Top panel: y-position of the lower lip midpoint (L_Y_) across frames in one run; bottom panel: x-position of the right mouth corner (RC_X_) in the same run. The trace is shown at the effective recording rate of 20 Hz.

**Fig. 2.**
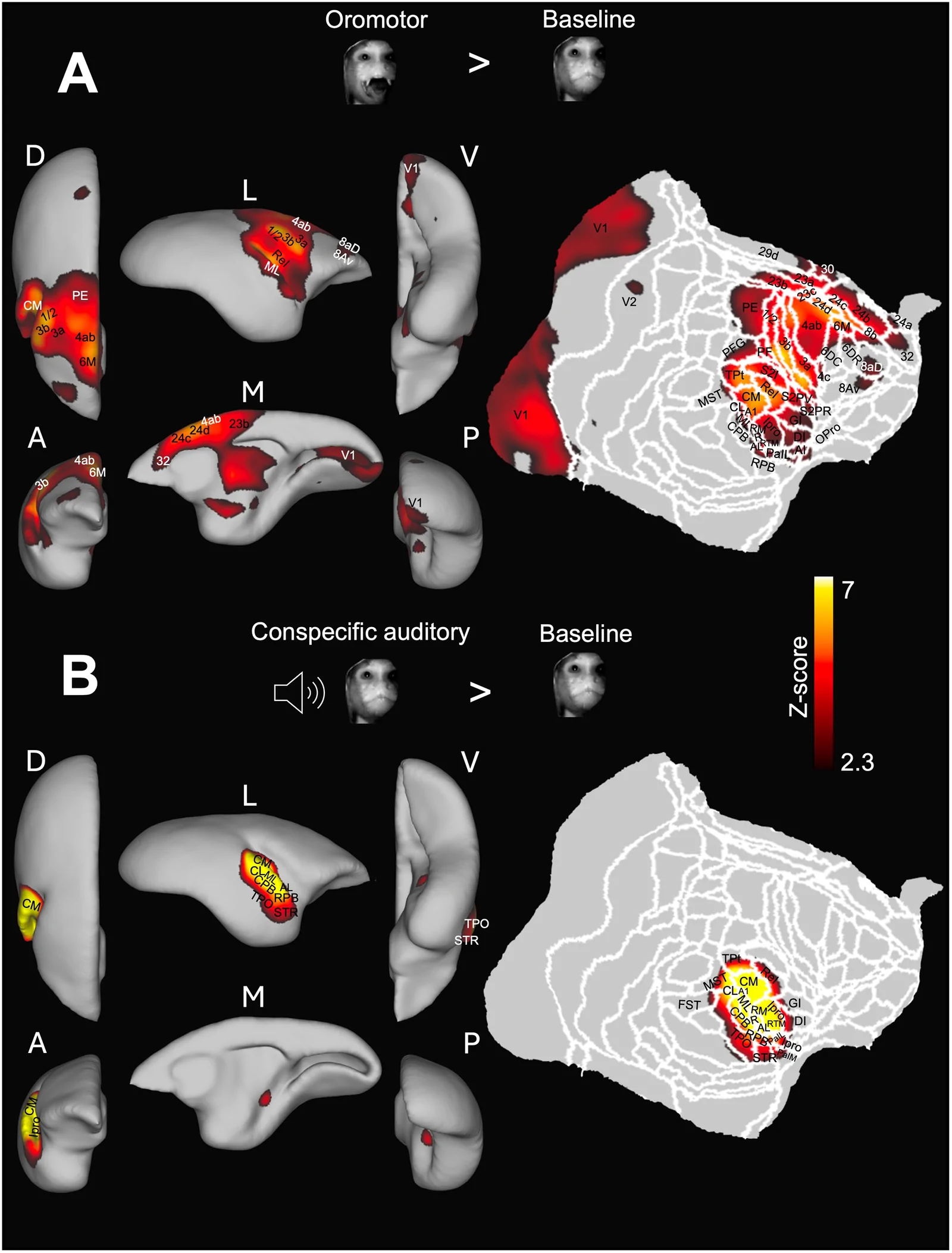
Cortical networks activated by oromotor movements and conspecific call listening. (A) Statistical activation maps for the oromotor movement condition (versus baseline) in the group of 11 awake marmosets. Activations are displayed on the right hemisphere’s fiducial cortical surface (views: dorsal “D”, ventral “V”, medial “M”, lateral “L”, anterior “A”, posterior “P”) and on a flattened cortical map. White outlines on the flatmap delineate cortical areas according to the Paxinos atlas (Paxinos et al., 2012), registered to the NIH marmoset brain template (Liu et al., 2018). (B) Cortical activation maps for the conspecific auditory condition (hearing calls vs. baseline), same format as A. Note the focal auditory cortex activations in contrast to the distributed activations in (A).

**Fig. 3.**
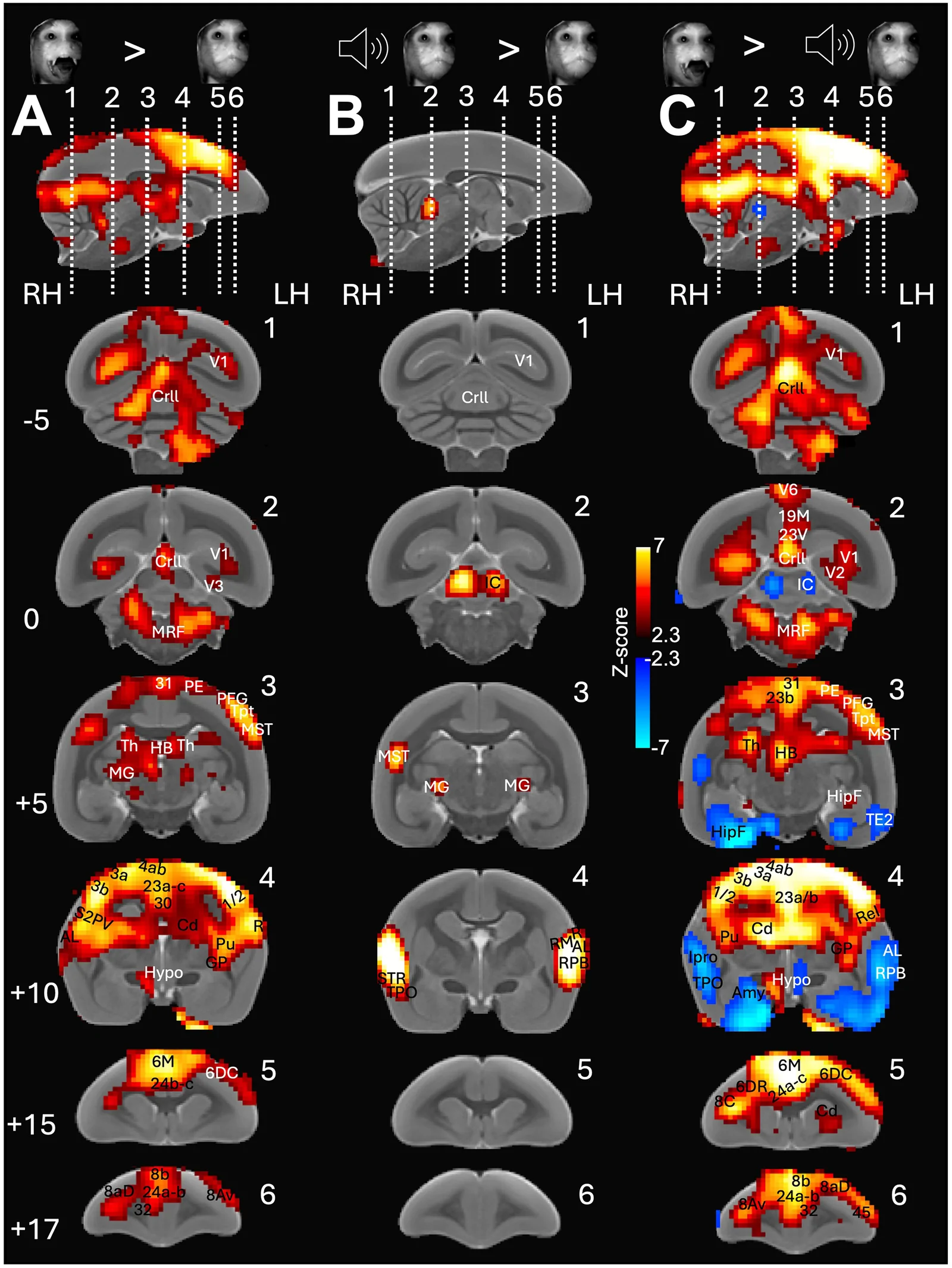
Volumetric activations for oromotor movements and conspecific auditory processing. (A) Group activation map for oromotor movements (vs. baseline) shown on representative sagittal and coronal slices of a high-resolution marmoset brain template (Liu et al., 2018) (with coronal slice locations indicated by intra-aural level in millimeter). The uppermost panel shows a mid-sagittal view highlighting midbrain/brainstem structure. Colored regions denote significant activation (*Z*-score scale). Key structures are labeled: Amy (amygdala), Crll (cerebellum), Cd (caudate), CM (caudal medial AC), MRF (medullary reticular formation), Th (thalamus), IC (inferior colliculus), Pu (putamen), GP (globus pallidus), MG (medial geniculate nucleus), HB (habenular nuclei), Hypo (hypothalamus), LH (left hemisphere), RH (right hemisphere). (B) Activation map for conspecific auditory (vs. baseline), in the same format. Note the prominent IC and MG activation, and relative lack of other subcortical activations. (C) Direct contrast of oromotor vs. conspecific auditory conditions. Warm colors denote regions more activated during oromotor movements; cool colors denote regions more activated during auditory processing. Subcortical structures with stronger oromotor activation (e.g., Crll, Th, basal ganglia, MRF, Hypo) are apparent.

Figure 2 presents group-level activation maps contrasting the oromotor and conspecific auditory (call-listening) conditions with baseline, for the right hemisphere. In the *oromotor > baseline* contrast (Fig. 2A), extensive activation was observed across both medial and lateral cortical regions. Strong BOLD responses were evident in the primary somatosensory cortex (SI)—including areas 3a, 3b, 1, and 2—and in secondary somatosensory regions, including the internal (S2I), parietal rostral (S2PR), and parietal ventral (S2PV) areas. Increased activity was also found within the primary motor cortex (area 4ab) and in premotor areas 6DC, 6DR, and 6M. Further activation extended into posterior parietal areas PE, PF, and PFG, as well as multiple insular fields—including agranular (AI), granular (GI), dysgranular (DI)—and the lateral part of the parainsular cortex (PaIL). Multiple auditory-related areas were also engaged, including the temporoparietal transitional area (Tpt) and retroinsular area (ReI), as well as several fields of the auditory cortex (AC)—including caudomedial (CM), caudolateral (CL), mediolateral (ML), anterolateral (AL), primary auditory (A1), rostral (R), rostromedial (RM), rostrotemporal (RT), rostrotemporal medial (RTM), and rostral parabelt (RPB). Additionally, caudal prefrontal areas 8aD, 8b, and 8Av, cingulate areas 23a–c, 24a–d, 30, and 32, as well as the primary visual cortex (V1), were activated during oromotor movements.

In contrast, the conspecific auditory (passive call-listening) condition (Fig. 2B) elicited a very different activation pattern, limited primarily to auditory-related cortices. Significant group-level activity was observed in AC fields, including CM, CL, ML, AL, A1, R, RM, RTM, RPB, and the caudal parabelt (CPB). Neighboring regions—such as Tpt, ReI, temporoparieto-occipital area (TPO), superior temporal rostral (STR), and medial superior temporal (MST)—were also significantly engaged in response to hearing conspecific calls. This overall spatial distribution of activations was bilateral, with comparable patterns observed in the left hemisphere (see Supplementary Figs. S2 and S3).

Subcortically, the oromotor condition also elicited strong activation in several regions, including the cerebellum (Crll), MG, thalamus (Th), habenular nuclei (HB), caudate (Cd), putamen (Pu), hypothalamus (Hypo), and globus pallidus (GP), as shown in Figure 3A. This subcortical activation profile differed sharply from that of the conspecific auditory condition, which showed robust activity largely restricted to IC and MG (Fig. 3B).

Our results also show that the oromotor condition evoked robust activation in midbrain regions—particularly the PAG, superior colliculus (SC), and pontine nuclei (PN) of the pons (Fig. 4A), and in the medullary reticular formation (MRF), as depicted in Figure 3A. In contrast, these regions showed no significant activity during the conspecific auditory condition (Figs. 3B and 4B).

**Fig. 4.**
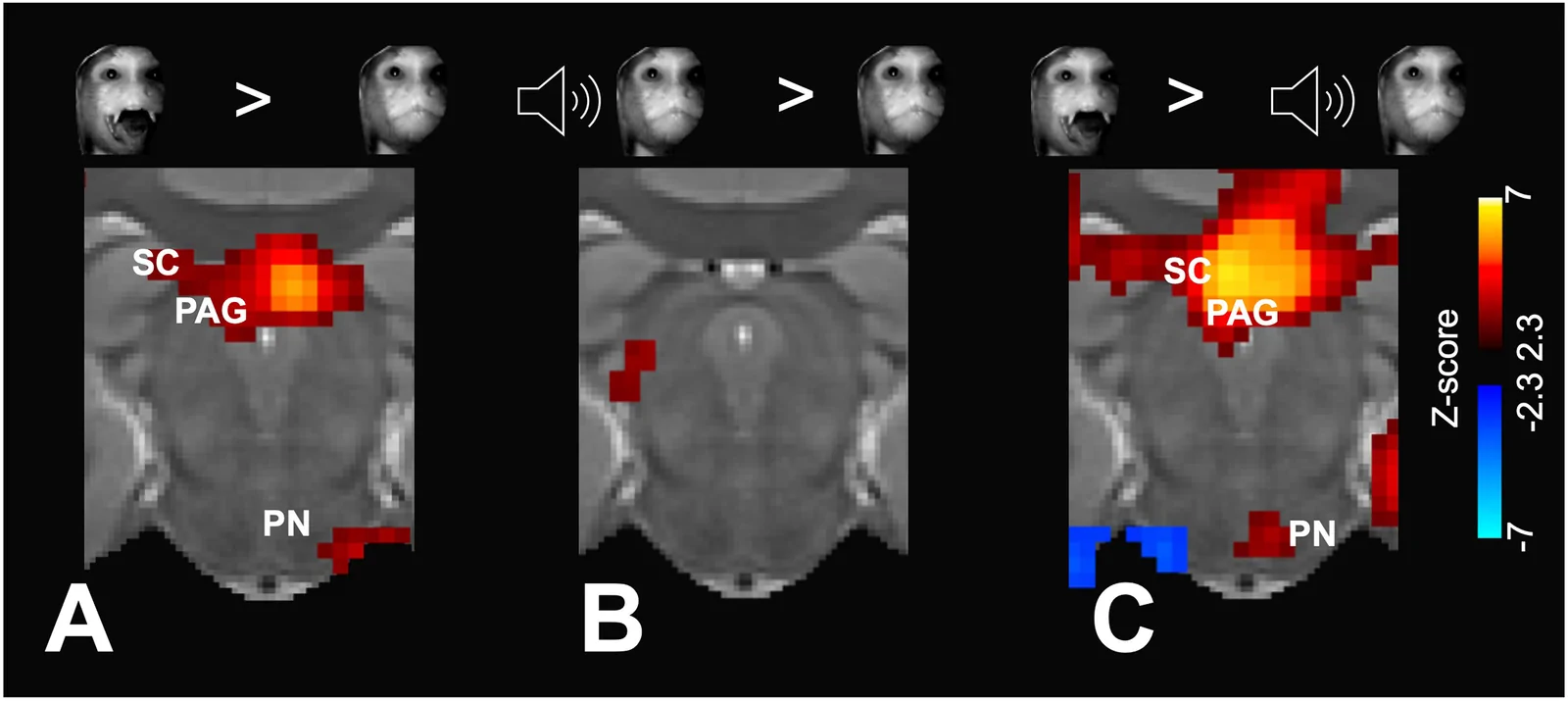
Midbrain and brainstem activations. Group activation at the intra-aural level of 3.8 mm, overlaid on a coronal slice of an anatomical template of the marmoset brain (Liu et al., 2018). (A) *Oromotor movements > baseline*. (B) *Conspecific auditory* > *baseline*. (C) *Oromotor movements > Conspecific auditory* contrast. The panels highlight midbrain structures: PAG (periaqueductal gray), SC (superior colliculus), PN (pontine nuclei). The oromotor condition clearly engages PAG, SC, and PN (A, C), whereas the auditory condition does not (B).

Figures 3C, 4C, and 5 display the direct contrast between the oromotor and conspecific auditory conditions, with regions showing greater oromotor-related activation represented in warm colors and those with higher activation during conspecific auditory processing in cool colors (on a *Z*-score scale). The results reveal broad activation during the oromotor condition across both medial and lateral cortical areas, with significant differences in several key regions. Medial prefrontal regions—including areas 32, 24a–d, and 23a–c—as well as caudal prefrontal regions 8C, 8b, 8aD, and 8Av exhibited stronger activation for oromotor movements relative to auditory processing (see Fig. 5). Similarly, premotor areas 6DC, 6DR, 6Va, and 6M and the primary motor cortex (4ab) displayed enhanced activation during oromotor movements compared with conspecific-call listening. This enhanced activity also extended into primary somatosensory regions (3a, 3b, 1, 2), secondary somatosensory areas (S2I, S2PR, S2PV), and parietal areas (PE, PF, PFG). Additionally, the contrast revealed larger activations in the SC, PAG, and PN (Fig. 4C), with stronger subcortical activation encompassing the Crll, Th, HB, Cd, Pu, GP, and Hypo (Fig. 3C).

**Fig. 5.**
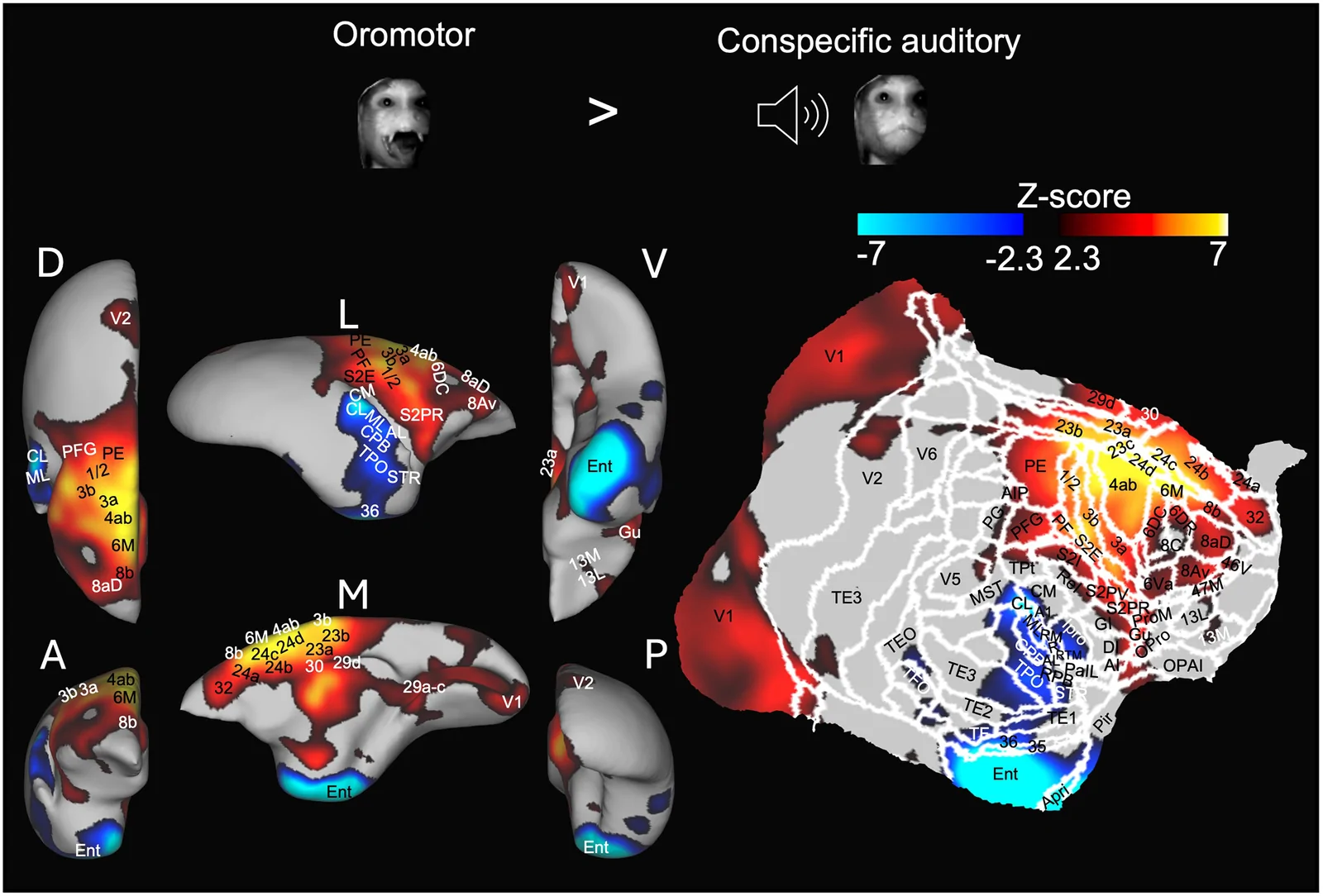
Cortical networks for oromotor vs. conspecific auditory. Group contrast maps (*oromotor movements* > *conspecific auditory*) displayed on the right hemisphere’s fiducial surface (views: D, V, M, L, A, P) and flatmap. Yellow–red indicate greater activation for oromotor; blue indicates greater activation for auditory events. The white outlines on the flatmap delineate cortical areas (Paxinos atlas (Paxinos et al., 2012) on the NIH template (Liu et al., 2018)). Marmoset oromotor movements induce stronger activation in motor, premotor, cingulate, and parietal regions, whereas auditory listening has higher activation mainly in auditory cortices (blue patches, largely in lateral belt areas).

Furthermore, cerebellar activation patterns differed markedly between the two conditions (Fig. 6). During oromotor movements versus baseline, robust positive BOLD responses were observed across the entire vermis—with peak intensity in lobules Va–d, VIa–d, and Xa–b—and extended into the hemispheres, including Crus I, Crus II, and the hemispheric portions of lobules V–VII (Fig. 6A). Unilateral negative BOLD responses were also observed in posterior regions, including lateral Crus I posterior and HVIIBa. In contrast, passive listening to conspecific calls vs. baseline elicited predominantly negative BOLD responses across broad regions of the cerebellum (Fig. 6B), with relatively no positive activation. Notably, the deactivation was not uniform across the cerebellum and was strongest in the vermis and medial hemispheric portions, broadly encompassing the regions engaged during oromotor movements (vermal lobules IV–VI and the hemispheric portions of Crus I and Crus II). The direct contrast between conditions (*oromotor > conspecific auditory*; Fig. 6C) localized greater cerebellar activation during oromotor movements to the vermis (specifically lobules IV–VI) and to Crus I and Crus II of the cerebellar hemispheres.

**Fig. 6.**
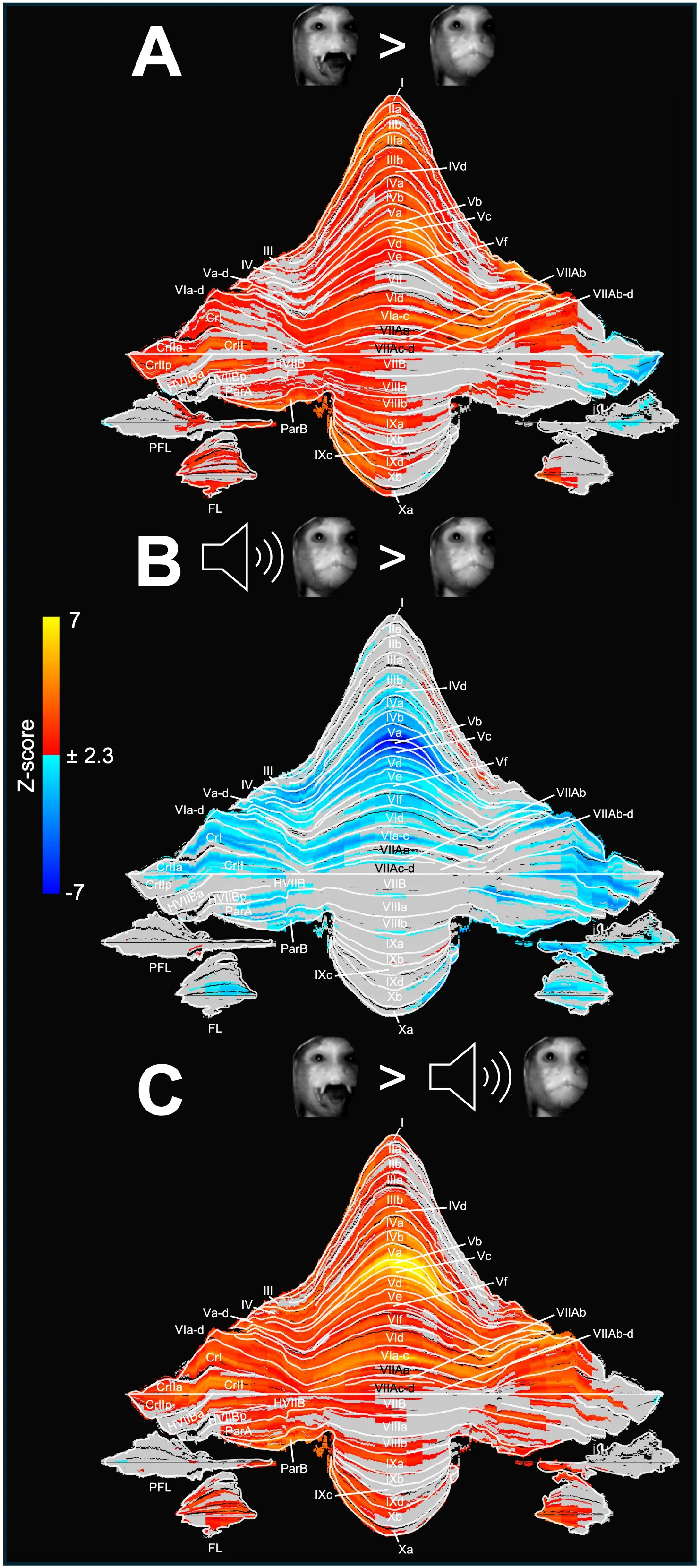
Cerebellar networks. Group activation maps projected on a flatmap of the marmoset cerebellum (Schaeffer et al., 2022). The flatmap representation unfolds the cerebellar cortex along each folium for visualization across lobules. White lines demarcate lobular boundaries (see online atlas for labels). (A) *Oromotor > baseline*. Robust positive BOLD activation (warm colors) is evident throughout the entire vermis, with peak intensity in lobules Va–d, VIa–d, and Xa–b, and extending into hemispheric regions. Negative BOLD responses (cool colors) are present unilaterally in lateral Crus I posterior and HVIIBa. (B) *Conspecific auditory > baseline*. Passive listening to conspecific calls elicited predominantly negative BOLD responses (cool colors) across broad regions of the cerebellum, with relatively no positive activation. (C) *Oromotor > conspecific auditory*. Warm colors indicate stronger activation during oromotor movements, whereas cool colors indicate stronger activation during call listening. The strongest differences are localized to the vermis (lobules IV–VI) and Crus I and Crus II.

To examine whether the acquired activation patterns depended on the threshold used to detect oromotor events, we repeated the analysis for the *oromotor > baseline* contrast using event sets defined at fixed thresholds of 2, 3.5, and 5 SD (Supplementary Fig. S4), spanning most of the 2–6 SD range used in the main analysis. The threshold of 6 SD was not included, as too few events were retained at that value for reliable estimation. This analysis was restricted to the 43 runs from 9 monkeys in which events were detected at all 3 thresholds. The number of events entering the analysis decreased from 1,332 at 2 SD to 870 at 3.5 SD and 648 at 5 SD. The main nodes of the oromotor network were present at all three thresholds, including the primary somatosensory (3a, 3b, 1/2) and primary motor (4ab) cortices, S2PV, AL and R, premotor area 6M, caudal prefrontal 8Av, cingulate areas 23a–c, 24b–c, and 31, and V1 at the cortical level, and the Cd, Pu, and GP, the Th and HB, the MRF, and the Crll at the subcortical level. The overall extent of significant activation decreased progressively as the threshold increased. Areas PE, Tpt, MST, MG, 6DC, and 32 showed significant activity only at 2 SD, whereas PFG, 30, 24a, 8aD, 8b, 8C, and 6Va were activated at 2 and 3.5 SD but not at 5 SD.

## 4 Discussion

This study provides, to the best of our knowledge, the first whole-brain mapping of the functional neural networks involved in oromotor movements in awake marmosets, demonstrating both cortical and subcortical activations associated with these functions. Our findings reveal a broadly distributed network of brain regions engaged during spontaneous movements of visible articulators. While both oromotor and conspecific auditory conditions activated some common regions—particularly within the core and belt fields of the auditory cortices and nearby temporoparietal areas—clear differences were evident in the broader activation maps, reflecting the distinct brain networks recruited by each task.

Similar to the results previously reported in marmosets (Dureux et al., 2024, 2025; Jafari et al., 2023, 2025), the conspecific auditory condition was primarily associated with activation in the auditory cortex, surrounding temporal areas, and auditory subcortical nuclei. In contrast, the oromotor conditions engaged a substantially broader network, including regions involved in sensorimotor integration, motor-related areas, somatosensory and cingulate cortices, prefrontal regions, and several subcortical areas. This overall pattern is consistent with human fMRI findings showing that movements of the lips, jaw, tongue, and larynx activate an overlapping cortical network encompassing premotor, primary motor, somatosensory, and parietal cortices, together with supplementary motor areas and inferior frontal and supramarginal gyri, as well as subcortical areas such as the putamen and cerebellar hemispheres (Grabski et al., 2012). These findings also support macaque work proposing an integrated face sensorimotor network linking medial and lateral motor regions with primary somatosensory cortex (Vázquez et al., 2025).

A closer look at the parietal cortex activated in our results reveals a pattern reflecting known effector-specific functional subdivisions. In macaques, area PF is engaged during mouth movements, PFG is linked to hand movements, and PE is involved in arm movements (Rizzolatti & Rozzi, 2018; Rozzi et al., 2008). Consistent with this functional organization, stimulation of the human inferior parietal lobule (IPL) can evoke the intention to move the contralateral hand, arm, foot, and lip or to talk (Desmurget et al., 2009). The activation we observed in PF, PFG, and PE during overt oromotor movements suggests that these parietal areas may be components of the underlying control system in marmosets. Areas PF, PE, and PFG in marmosets display strong resting-state functional connectivity with premotor area 6DC (Hori et al., 2022), which we found activated for the specified movements in this study. This supports the idea that the parietal regions, together with their premotor connections, form a circuit contributing to the integration of motor commands and sensory feedback necessary for oromotor functions. A potential confounding factor, however, is that we monitored only the face and not the rest of the body. Although facial movements were reliably captured on video, and the animals were head fixed and restrained in a sphinx position to minimize gross motion, we cannot fully exclude that small, co-occurring limb or postural movements contributed to part of the parietal and sensorimotor activation. However, since event onsets were derived solely from facial landmarks, only body movements coinciding with a detected oromotor event could have contributed to these maps; movements occurring at other times were not captured by the model. As these body movements were not tracked, their contribution cannot be quantified, and the parietal and sensorimotor responses reported here should be interpreted as reflecting oromotor events together with any incidental body movements that accompanied them.

Frontal motor regions also showed significant engagement during oromotor events, with significant recruitment of the medial (6M) and dorsal (6DR, 6DC) premotor cortices. This pattern is consistent with an increased *cFos* expression selectively within dorsal premotor areas during marmoset vocalizations (Miller et al., 2010). Electrophysiological recordings in marmosets have further shown that premotor cortex neurons are modulated during self-initiated vocalizations, with some neurons selective for vocal production and others differentiating between vocalization and non-vocal oromotor movements such as licking (Roy et al., 2016). In addition, call-type-specific activity has been reported in frontal cortex neurons during natural vocal exchanges (Zhao & Wang, 2023). Together, these findings support a key role for dorsal premotor cortex in marmoset oromotor control.

Beyond these parietal and premotor contributions, the anterior cingulate cortex also played a central role in oromotor conditions. Significant activation was observed in areas 24 and 32 during oromotor movements, mirroring findings from West and Larson’s single-unit recordings in awake macaques (West & Larson, 1995). They showed that neurons in the mesial cortex, located along the anterior cingulate sulcus, display robust firing patterns during oromotor behaviors, with distinct populations selectively responsive to only vocalization, only jaw movements, both behaviors, and other non-vocal oromotor activities such as lip movements (West & Larson, 1995). Our fMRI results, while at a larger scale, are consistent with this fine-grained electrophysiological evidence, supporting that the ACC acts as a major hub for integrating motivational, cognitive, and motor signals for oromotor actions.

In addition to ACC involvement, prefrontal regions were also recruited—particularly the lateral prefrontal area 8aD. Prefrontal areas are typically associated with executive processes such as planning, decision making, and working memory in primates (Miller & Cohen, 2001), suggesting that even spontaneous oromotor movements in marmosets may involve some degree of higher-order control (e.g., deciding when to vocalize or move articulators). The engagement of both ACC and lateral prefrontal regions is consistent with long-standing models in nonhuman primates in which prefrontal–cingulate circuits contribute to the initiation and regulation of vocal production—one of the oromotor behaviors observed in the present study (Jürgens, 1983, 2009; Jürgens & Pratt, 1979a, 1979b; Kirzinger & Jürgens, 1982; Müller-Preuss & Jürgens, 1976; Von Cramon & Jürgens, 1983).

However, we did not observe a clear recruitment of the ventral premotor cortex (area 6Va) in the *oromotor > baseline* contrast in the main analysis. Although 6Va appeared in the direct *oromotor > conspecific auditory* comparison, this effect may partly reflect reduced activity in motor-related regions during passive call listening (Jafari et al., 2025) rather than strong ventral frontal engagement during spontaneous oromotor events. In macaques, the ventrolateral frontal regions (PMv/vlPFC)—proposed as a putative homolog of human area 44 within the Broca-related speech network (Friederici, 2011; Petrides et al., 2005)—control orofacial musculatures and likely mediated higher-order control of orofacial and communicative behaviors before acquiring, in humans, a more specialized role in the temporal coordination of speech rather than in direct articulatory control (Hage & Nieder, 2016; Loh et al., 2020; Long et al., 2016). Consistent with this functional distinction, cooling Broca’s region in awake neurosurgical patients altered speech timing, whereas cooling the ventral precentral gyrus altered articulation, supporting distinct roles in motor sequencing and execution (Long et al., 2016). The temporal-sequencing functions attributed to ventral frontal cortex are not strongly engaged by the spontaneous oromotor events recorded here in marmosets; therefore, the limited ventral frontal recruitment we observe is expected. Moreover, neurons of these regions fire specifically during conditioned (voluntary) vocalizations but not during spontaneous vocal production (Coudé et al., 2011; Hage & Nieder, 2013), which may explain why our spontaneous events primarily recruited dorsomedial frontal nodes. Immunohistochemical mapping of spontaneous vocal production in freely behaving marmosets has, however, shown recruitment of ventrolateral frontal regions (Simões et al., 2010). This discrepancy with our results may reflect differences in task context (Loh et al., 2020) and behavioral state between freely behaving animals and restrained fMRI conditions employed in this study.

A parallel distinction is also well established for non-vocal oromotor movements. In primates, the lateral motor pathway (M1, ventrolateral premotor cortex) preferentially mediates voluntary, goal-directed facial and oromotor movements, whereas mid-cingulate motor areas contribute substantially to spontaneous and emotional facial expressions (Morecraft et al., 2001; Müri, 2016; Vázquez et al., 2025). The awake-restrained scanning environment used in the current study—head fixation, novel auditory stimuli, and the absence of behavioral targets such as food—likely biased the detected oromotor events toward spontaneous and reactive forms rather than goal-directed actions. The pattern observed here, with prominent anterior cingulate engagement and limited ventral premotor recruitment, is consistent with this behavioral context; paradigms eliciting voluntary, goal-directed oromotor behaviors—such as learned vocal tasks or microstimulation studies (Miller et al., 2015; Petrides et al., 2005)—would be expected to recruit ventral and lateral frontal regions more strongly. This pattern is consistent with a predominantly dorsal frontal architecture for the spontaneous oromotor behaviors sampled in this study.

Primary motor and somatosensory cortices were likewise strongly engaged. Although chewing and swallowing were once considered to be governed exclusively by brainstem central pattern generators, it is now widely recognized that M1 also contributes to these semi-automatic oromotor behaviors, as well as to fully voluntary actions such as speech and vocalization (Avivi-Arber et al., 2011; Hamdy et al., 1999; Sahara et al., 2012; Sessle et al., 2005, 2007). Moreover, the face representation of M1 relies on sensory input from corresponding somatosensory areas in SI to shape and modulate these behaviors (Avivi-Arber et al., 2011; Sahara et al., 2012; Sessle et al., 2005). The robust engagement of areas 4ab, 3a, 3b, 1, and 2 during the oromotor condition aligns closely with this model and underscores that cortical sensorimotor circuits are integral to executing jaw/tongue/lip movements in marmosets.

Beyond M1 and SI, secondary somatosensory areas (S2PV, S2PR) were also engaged during oromotor conditions. Detailed electrophysiological mapping in macaques has shown that the lateral sulcus contains the second somatosensory area (SII) and the parietal ventral area (PV), which share a common boundary at the representation of the lips and mouth (Krubitzer et al., 1995). The strong activity we observed in S2PV and S2PR aligns well with this organization, highlighting their roles in integrating tactile and proprioceptive feedback during oromotor actions.

Alongside these motor, premotor, and somatosensory activations, sensory cortical areas also showed significant activity. We observed enhanced activation within auditory cortical fields during the oromotor condition (relative to baseline). This may reflect the processing of a predictive corollary discharge signal from the motor system, which anticipates the sensory consequences of imminent oromotor actions and helps maintain sensitivity to the external acoustic environment (Eliades & Wang, 2003; Paus et al., 1996; Schneider et al., 2014). Consistent with this interpretation, recent simultaneous recordings in marmosets have demonstrated directed frontal-to-auditory signaling in the pre-vocal period and bidirectional interactions during altered vocal feedback that predict compensatory vocal adjustments (Tsunada & Eliades, 2025). Despite this engagement of auditory areas during self-generated movements, auditory cortical activity was markedly weaker during oromotor movements than during the conspecific auditory condition. This reduced responsiveness aligns with the well-established view that auditory cortical responses are attenuated during active movement (such as vocalization) and are modulated by speech-related motor activity in both humans and nonhuman primates (Eliades & Wang, 2003; Paus et al., 1996; Schneider et al., 2014).

In addition to auditory effects, the primary visual cortex (V1) also showed significant activation during the oromotor condition. This is likely caused by natural concomitant behaviors (e.g., eyelid openings and pupil dilations that often accompany oromotor movements) rather than by visual processing *per se*, since no visual stimuli were presented.

At the brainstem level, several nuclei were also engaged during the oromotor condition. The strong activation of the medullary reticular formation in these events is consistent with evidence that this region contains key components of the brainstem central pattern generators responsible for rhythmic oromotor functions, including jaw movement, licking, and chewing (Moore et al., 2014; Travers et al., 1997). The periaqueductal gray has been implicated in vocal production in primates, acting as a relay between cingulate cortex and brainstem premotor centers (Dujardin & Jürgens, 2005, 2006; Düsterhöft et al., 2004; Hage & Nieder, 2016; Jürgens, 2009; Siebert & Jürgens, 2003), but it also participates in a broad range of autonomic, nociceptive, and non-vocal oromotor functions (Bandler & Shipley, 1994; Benarroch, 2012; Zhang et al., 2024). The engagement of PAG, PN (pons), and MRF in our results is, therefore, aligned with the recruitment of brainstem circuits supporting the vocal and non-vocal oromotor repertoire. Although video and microphone pilot recordings indicated that a limited number of oromotor events were accompanied by vocalization (see Figs. 1A and B), future studies incorporating simultaneous in-scanner audio recording with event-by-event behavioral classification will be required to attribute specific brainstem activations to vocal versus non-vocal events.

In addition to the brainstem, several subcortical structures were also involved in the control of the oromotor activities. In our results, oromotor movements strongly activated multiple basal ganglia nuclei, the superior colliculus, the thalamus, the hypothalamus, and the cerebellar vermis and hemispheres. These findings are aligned with evidence that the basal ganglia (caudate, putamen, globus pallidus), thalamus, and cerebellum are known to modulate and refine oromotor behaviors (Avivi-Arber et al., 2011; Grabski et al., 2012; Sahara et al., 2012; Sessle et al., 2005).

Moreover, the observed differences in cerebellar activation between oromotor and conspecific auditory tasks provide further insight into the complex neural coordination underlying these movements. Oromotor functions significantly engaged regions along the vermis of the cerebellum, suggesting a role in integrating autonomic or affective responses with peripheral motor systems (ranging from the diaphragm to the larynx) to produce coherent oromotor expressions (Ceravolo et al., 2021; Jürgens, 2009). Consistent with this cerebellar involvement, a recent study in marmosets also demonstrated that cerebellar circuits contribute to precise tongue control during goal-directed licking (Hage et al., 2025). In contrast, listening to conspecific calls showed no comparable positive cerebellar activation, emphasizing a clear dissociation between motor and non-motor processes. Indeed, the conspecific auditory condition was associated with predominantly negative BOLD responses across broad regions of the cerebellum. This pattern is consistent with our previous observation in marmosets, where similar cerebellar deactivation during passive call listening was interpreted as reflecting a reduction in minor spontaneous body and limb movements as the animals enter a state of stillness to attend to the auditory stimuli (Jafari et al., 2025). Notably, the deactivation during listening to calls extended across most of the cerebellar regions that were significantly engaged during oromotor movements, consistent with this interpretation of motor disengagement during attentive listening. Thus, the direct contrast between oromotor and auditory conditions reflects both the positive activation of cerebellar regions during overt movement events and the relative suppression of cerebellar activity during listening to conspecific calls.

The concurrent engagement of the superior colliculus, hypothalamus, and PAG is also notable in our findings. Sudden, unexpected threat in proximity has been shown to recruit the medial hypothalamus in marmosets, encoding an internal threat state. In the same species, the ventromedial hypothalamus projects to the dorsal PAG—involved in defensive responses such as freezing—and is anatomically connected with the superior colliculus (Montardy et al., 2021). Hypothalamic engagement has also been reported in awake head-fixed marmosets viewing negative facial expressions, accompanied by an increase in respiration rate and interpreted as reflecting the integration of autonomic and arousal responses (Dureux et al., 2023). A subset of the events sampled here (92 of 2,033) involved visible discomfort or alarm expressions, and although all events were modeled with a single regressor and their individual contributions cannot be quantified, engagement of this system during those events is plausible. The superior colliculus and PAG may, therefore, contribute to the present maps in more than one capacity. Overall, our brainstem and subcortical findings support the view that while core brainstem circuits generate oromotor movement patterns, they are modulated by higher-order structures—including cortical, cerebellar, basal ganglia, and superior colliculus pathways—to refine and coordinate motor outputs (Bowman & Aldes, 1980; Moore et al., 2014).

A methodological consideration in using deep learning and markerless pose estimation to define behavioral events in this study concerns how those oromotor events are detected from the tracked traces. Because the onsets of the events define the regressors for the GLM analysis, the choice of threshold for detecting the events could in principle impact the resulting activation maps. However, we took two steps to limit this. First, the final event list was not determined by the threshold alone, and each run was reviewed in full against its corresponding video, so that false detections were removed and the actual movements missed by the threshold were added to the final list in our main analysis. Second, we repeated the analysis using event sets acquired by the fixed thresholds of 2, 3.5, and 5 SD, and the main regions of the network—sensorimotor, cingulate, subcortical, and brainstem—were present at every threshold. However, the spatial extent of the activation decreased progressively as the threshold increased. Higher thresholds retained only larger movements, reducing the number of events entering the GLM model (from 1,332 to 648 for 2 SD and 5 SD, respectively) and potentially contributing to the less extensive functional network observed at higher thresholds. The areas detected only at the lower thresholds, including the posterior parietal areas PE and PFG and ventral premotor corte x 6Va, were, therefore, not necessarily absent at 5 SD. The consistency of the core network across thresholds indicates that the overall pattern reported here does not depend on the particular threshold applied.

All oromotor events were entered as impulses at their onset and were not characterized by their duration or kinematic structure, so any variability in these features was not captured by the statistical modeling. The traces acquired from DeepLabCut keep this information, and future work could model event duration or movement amplitude explicitly to test whether these features influence the BOLD response.

A further consideration in this study concerns the contribution of physiological noise, particularly to subcortical and brainstem signals. Awake marmosets breathe at roughly 78 breaths per minute (~1.3 Hz; Bishop et al., 2022), and heart rate is on the order of 150–250 beats per minute under anesthesia (~2.5–4.2 Hz; Moussavi et al., 2020), with higher rates expected in the awake state. Because each volume was acquired every 3 seconds (Nyquist frequency ≈0.167 Hz), both rhythms lie well above the highest frequency the acquisition can represent and are, therefore, aliased into lower frequencies rather than removed by the temporal filtering applied during preprocessing steps (see Section 2). A residual physiological contribution to the subcortical and brainstem regions, therefore, cannot be fully excluded. Two features of the present design nonetheless limit this concern. First, the oromotor events occurred at irregular intervals, so aliased cardiac and respiratory rhythms were unlikely to consistently coincide with the modeled event onsets. Second, the activations reported here were anatomically circumscribed and consistent with those described in our previous marmoset fMRI studies using the same acquisition protocol (Jafari et al., 2025). If physiological noise were the main contributor to the maps, a more diffuse pattern would be expected.

Taken together, the results of this study suggest that spontaneous oromotor control in marmosets recruits a distributed network spanning brainstem, subcortical, and cortical structures, with a predominantly dorsal frontal organization and prominent posterior parietal engagement. Because continuous audio was not recorded during the main sessions, vocal and non-vocal events were modeled with a single regressor; the predominantly dorsal pattern described here should, therefore, be interpreted as applying to the combined set of spontaneous oromotor events. The possibility that vocal and non-vocal events recruit partly distinct circuits cannot, therefore, be excluded with the present data. One anatomical factor that may shape vocal–motor organization across primates is cortico-laryngeal connectivity. Anterograde tracing from the macaque laryngeal motor cortex has shown that descending projections do not reach laryngeal motoneurons directly—a connection that is present in humans and has been proposed as a substrate for volitional voice control and speech evolution (Simonyan & Jürgens, 2003). Characterizing the oromotor network in a New World primate, therefore, provides a comparative anchor for understanding its organization across species and how rhythmic oromotor systems of this kind may relate to the evolutionary origins of human speech.

## Supplementary Material

Supplementary Material

## Data Availability

The group-level and subject-level functional maps supporting this study, together with the analysis codes and auditory stimuli, are publicly available on Zenodo (https://doi.org/10.5281/zenodo.22259741 and https://doi.org/10.5281/zenodo.22261124, respectively).

## References

[IMAG.a.1419-b1] Audacity, T. (2017). Audacity. The Name Audacity (R) Is a Registered Trademark of Dominic Mazzoni. http://audacity.sourceforge.net/help/faq?s=install&item=lame-mp3

[IMAG.a.1419-b2] Avants, B. B., Tustison, N., & Song, G. (2009). Advanced Normalization Tools (ANTS). Insight Journal, 2(365), 1–35. https://brianavants.wordpress.com/2012/04/13/updated-ants-compile-instructions-april-12-2012/

[IMAG.a.1419-b3] Avivi-Arber, L., Martin, R., Lee, J. C., & Sessle, B. J. (2011). Face sensorimotor cortex and its neuroplasticity related to orofacial sensorimotor functions. Archives of Oral Biology, 56(12), 1440–1465. 10.1016/j.archoralbio.2011.04.005 21550585

[IMAG.a.1419-b4] Bandler, R., & Shipley, M. T. (1994). Columnar organization in the midbrain periaqueductal gray: Modules for emotional expression? Trends in Neurosciences, 17(9), 379–389. 10.1016/0166-2236(94)90047-7 7817403

[IMAG.a.1419-b5] Benarroch, E. E. (2012). Periaqueductal gray: An interface for behavioral control. Neurology, 78(3), 210–217. 10.1212/wnl.0b013e31823fcdee 22249496

[IMAG.a.1419-b6] Bergman, T. J. (2013). Speech-like vocalized lip-smacking in geladas. Current Biology, 23(7), R268–R269. 10.1016/j.cub.2013.02.036 23578870

[IMAG.a.1419-b7] Bishop, M., Weinhold, M., Turk, A. Z., Adeck, A., & Sheikhbahaei, S. (2022). An open-source tool for automated analysis of breathing behaviors in common marmosets and rodents. ELife, 11, e71647. 10.7554/eLife.71647 PMC8856653 35049499

[IMAG.a.1419-b8] Bowman, J. P., & Aldes, L. D. (1980). Organization of the cerebellar tongue representation in the monkey. Experimental Brain Research, 39(3), 249–259. 10.1007/bf00237114 6772461

[IMAG.a.1419-b9] Bradski, G. (2000). The OpenCV Library. Dr. Dobb’s Journal of Software Tools, 25(11), 120–125. https://jacobfilipp.com/DrDobbs/articles/DDJ/2000/0011/0011k/0011k.htm

[IMAG.a.1419-b10] Ceravolo, L., Frühholz, S., Pierce, J., Grandjean, D., & Péron, J. (2021). Basal ganglia and cerebellum contributions to vocal emotion processing as revealed by high-resolution fMRI. Scientific Reports, 11(1), 10645. 10.1038/s41598-021-90222-6 PMC8138027 34017050

[IMAG.a.1419-b11] Cerkevich, C. M., Rathelot, J.-A., & Strick, P. L. (2022). Cortical basis for skilled vocalization. Proceedings of the National Academy of Sciences of the United States of America, 119(19), e2122345119. 10.1073/pnas.2122345119 PMC9171651 35507879

[IMAG.a.1419-b12] Correia-Caeiro, C., Burrows, A., Wilson, D. A., Abdelrahman, A., & Miyabe-Nishiwaki, T. (2022). CalliFACS: The common marmoset Facial Action Coding System. PLoS One, 17(5), e0266442. 10.1371/journal.pone.0266442 PMC9113598 35580128

[IMAG.a.1419-b13] Coudé, G., Ferrari, P. F., Rodà, F., Maranesi, M., Borelli, E., Veroni, V., Monti, F., Rozzi, S., & Fogassi, L. (2011). Neurons controlling voluntary vocalization in the macaque ventral premotor cortex. PLoS One, 6(11), e26822. 10.1371/journal.pone.0026822 PMC3206851 22073201

[IMAG.a.1419-b14] Cox, R. W. (1996). AFNI: Software for analysis and visualization of functional magnetic resonance neuroimages. Computers and Biomedical Research, 29(3), 162–173. 10.1006/cbmr.1996.0014 8812068

[IMAG.a.1419-b15] Culjak, I., Abram, D., Pribanic, T., Dzapo, H., & Cifrek, M. (2012). A brief introduction to OpenCV. In Proceedings of the 35th International Convention MIPRO (pp. 1725–1730). IEEE. https://ieeexplore.ieee.org/document/6240859

[IMAG.a.1419-b16] Desmurget, M., Reilly, K. T., Richard, N., Szathmari, A., Mottolese, C., & Sirigu, A. (2009). Movement intention after parietal cortex stimulation in humans. Science, 324(5928), 811–813. 10.1126/science.1169896 19423830

[IMAG.a.1419-b17] Dujardin, E., & Jürgens, U. (2005). Afferents of vocalization-controlling periaqueductal regions in the squirrel monkey. Brain Research, 1034(1–2), 114–131. 10.1016/j.brainres.2004.11.048 15713263

[IMAG.a.1419-b18] Dujardin, E., & Jürgens, U. (2006). Call type-specific differences in vocalization-related afferents to the periaqueductal gray of squirrel monkeys (*Saimiri sciureus*). Behavioural Brain Research, 168(1), 23–36. 10.1016/j.bbr.2005.10.006 16297458

[IMAG.a.1419-b19] Dureux, A., Zanini, A., & Everling, S. (2023). Face-selective patches in marmosets are involved in dynamic and static facial expression processing. Journal of Neuroscience, 43(19), 3477–3494. 10.1523/JNEUROSCI.1484-22.2023 PMC10184744 37001990

[IMAG.a.1419-b20] Dureux, A., Zanini, A., & Everling, S. (2024). Mapping of facial and vocal processing in common marmosets with ultra-high field fMRI. Communications Biology, 7(1), 317. 10.1038/s42003-024-06002-1 PMC10937914 38480875

[IMAG.a.1419-b21] Dureux, A., Zanini, A., Trapeau, R., Belin, P., & Everling, S. (2025). Functional organization of voice patches in marmosets and cross-species comparisons with macaques and humans. Current Biology, 35(16), 3869.e4–3882.e4. 10.1016/j.cub.2025.07.008 40712583

[IMAG.a.1419-b22] Düsterhöft, F., Häusler, U., & Jürgens, U. (2004). Neuronal activity in the periaqueductal gray and bordering structures during vocal communication in the squirrel monkey. Neuroscience, 123(1), 53–60. 10.1016/j.neuroscience.2003.07.007 14667441

[IMAG.a.1419-b23] Eliades, S. J., & Miller, C. T. (2017). Marmoset vocal communication: Behavior and neurobiology. Developmental Neurobiology, 77(3), 286–299. 10.1002/dneu.22464 27739195

[IMAG.a.1419-b24] Eliades, S. J., & Wang, X. (2003). Sensory-motor interaction in the primate auditory cortex during self-initiated vocalizations. Journal of Neurophysiology, 89(4), 2194–2207. 10.1152/jn.00627.2002 12612021

[IMAG.a.1419-b25] Friederici, A. D. (2011). The brain basis of language processing: From structure to function. Physiological Reviews, 91(4), 1357–1392. 10.1152/physrev.00006.2011.-Lan 22013214

[IMAG.a.1419-b26] Ghazanfar, A. A., & Takahashi, D. Y. (2014). The evolution of speech: Vision, rhythm, cooperation. Trends in Cognitive Sciences, 18(10), 543–553. 10.1016/j.tics.2014.06.004 PMC4177957 25048821

[IMAG.a.1419-b27] Gilbert, K. M., Dureux, A., Jafari, A., Zanini, A., Zeman, P., Menon, R. S., & Everling, S. (2023). A radiofrequency coil to facilitate task-based fMRI of awake marmosets. Journal of Neuroscience Methods, 383, 109737. 10.1016/j.jneumeth.2022.109737 36341968

[IMAG.a.1419-b28] Grabski, K., Lamalle, L., Vilain, C., Schwartz, J. L., Vallée, N., Tropres, I., Baciu, M., Le Bas, J. F., & Sato, M. (2012). Functional MRI assessment of orofacial articulators: Neural correlates of lip, jaw, larynx, and tongue movements. Human Brain Mapping, 33(10), 2306–2321. 10.1002/hbm.21363 PMC6870116 21826760

[IMAG.a.1419-b29] Hage, P., Fakharian, M. A., Shoup, A. M., Pi, J. S., Sedaghat-Nejad, E., Orozco, S. P., Jang, I. K., Looi, V., Elseweifi, H. Y., Mohammadrezaei, N., Vasserman, A. N., Arginteanu, T., & Shadmehr, R. (2025). Control of tongue movements by the Purkinje cells of the cerebellum. PLoS Biology, 23(4), e3003110. 10.1101/2024.07.25.604757 PMC11984719 40208864

[IMAG.a.1419-b30] Hage, S. R. (2009). Neuronal networks involved in the generation of vocalization. In S. M. Brudzynski (Ed.), Handbook of mammalian vocalization: An integrative neuroscience approach (Vol. 19, 8.3). Academic Press. 10.1016/b978-0-12-374593-4.00032-2

[IMAG.a.1419-b31] Hage, S. R., & Nieder, A. (2013). Single neurons in monkey prefrontal cortex encode volitional initiation of vocalizations. Nature Communications, 4(1), 2409. 10.1038/ncomms3409 24008252

[IMAG.a.1419-b32] Hage, S. R., & Nieder, A. (2016). Dual neural network model for the evolution of speech and language. Trends in Neurosciences, 39(12), 813–829. 10.1016/j.tins.2016.10.006 27884462

[IMAG.a.1419-b33] Hamdy, S., Mikulis, D. J., Crawley, A., Xue, S., Lau, H., Henry, S., & Diamant, N. E. (1999). Cortical activation during human volitional swallowing: An event-related fMRI study. American Journal of Physiology-Gastrointestinal and Liver Physiology, 277(1), G219-G225. 10.1152/ajpgi.1999.277.1.g219 10409170

[IMAG.a.1419-b34] Hauser, M. D., & Ybarra, M. S. (1994). The role of lip configuration in monkey vocalizations: Experiments using xylocaine as a nerve block. Brain and Language, 46(2), 232–244. 10.1006/brln.1994.1014 8137144

[IMAG.a.1419-b35] Hori, Y., Clery, J. C., Schaeffer, D. J., Menon, R. S., & Everling, S. (2022). Functional organization of frontoparietal cortex in the marmoset investigated with awake resting-state fMRI. Cerebral Cortex, 32(9), 1965–1977. 10.1093/cercor/bhab328 PMC9070335 34515315

[IMAG.a.1419-b36] Jafari, A., Dureux, A., Zanini, A., Menon, R. S., Gilbert, K. M., & Everling, S. (2023). A vocalization-processing network in marmosets. Cell Reports, 42(5), 112526. 10.1016/j.celrep.2023.112526 37195863

[IMAG.a.1419-b37] Jafari, A., Dureux, A., Zanini, A., Menon, R. S., Gilbert, K. M., & Everling, S. (2025). Unique cortical and subcortical activation patterns for different conspecific calls in marmosets. Journal of Neuroscience, 45(3), e0670242024. 10.1523/JNEUROSCI.0670-24.2024 PMC11735661 39516045

[IMAG.a.1419-b38] Jürgens, U. (1983). Afferent fibers to the cingular vocalization region in the squirrel monkey. Experimental Neurology, 80(2), 395–409. 10.1016/0014-4886(83)90291-1 6840246

[IMAG.a.1419-b39] Jürgens, U. (2002). Neural pathways underlying vocal control. Neuroscience & Biobehavioral Reviews, 26(2), 235–258. 10.1016/s0149-7634(01)00068-9 11856561

[IMAG.a.1419-b40] Jürgens, U. (2009). The neural control of vocalization in mammals: A review. Journal of Voice, 23(1), 1–10. 10.1016/j.jvoice.2007.07.005 18207362

[IMAG.a.1419-b41] Jürgens, U., Maurus, M., Ploog, D., & Winter, P. (1967). Vocalization in the squirrel monkey (*Saimiri sciureus*) elicited by brain stimulation. Experimental Brain Research, 4(2), 114–117. 10.1007/bf00240356 4970751

[IMAG.a.1419-b42] Jürgens, U., & Pratt, R. (1979a). Role of the periaqueductal grey in vocal expression of emotion. Brain Research, 167(2), 367–378. 10.1016/0006-8993(79)90830-8 109167

[IMAG.a.1419-b43] Jürgens, U., & Pratt, R. (1979b). The cingular vocalization pathway in the squirrel monkey. Experimental Brain Research, 34(3), 499–510. 10.1007/bf00239145 105921

[IMAG.a.1419-b44] Kent, R. D. (2015). Nonspeech oral movements and oral motor disorders: A narrative review. American Journal of Speech-Language Pathology, 24(4), 763–789. 10.1044/2015_AJSLP-14-0179 PMC4698470 26126128

[IMAG.a.1419-b45] Kirzinger, A., & Jürgens, U. (1982). Cortical lesion effects and vocalization in the squirrel monkey. Brain Research, 233(2), 299–315. 10.1016/0006-8993(82)91204-5 7059813

[IMAG.a.1419-b46] Krubitzer, L., Clarey, J., Tweedale, R., Elston, G., & Calford, M. (1995). A redefinition of somatosensory areas in the lateral sulcus of macaque monkeys. The Journal of Neuroscience, 15(5), 3821–3839. 10.1523/jneurosci.15-05-03821.1995 PMC6578217 7751949

[IMAG.a.1419-b47] Larson, C. R., & Kistler, M. K. (1986). The relationship of periaqueductal gray neurons to vocalization and laryngeal EMG in the behaving monkey. Experimental Brain Research, 63(3), 596–606. 10.1007/bf00237482 3758271

[IMAG.a.1419-b48] Lindblom, B. E. F., & Sundberg, J. E. F. (1971). Acoustical consequences of lip, tongue, jaw, and larynx movement. Journal of the Acoustical Society of America, 50(4), 1166–1179. http://asadl.org/terms 10.1121/1.1912750 5117649

[IMAG.a.1419-b49] Liu, C., Ye, F. Q., Yen, C. C. C., Newman, J. D., Glen, D., Leopold, D. A., & Silva, A. C. (2018). A digital 3D atlas of the marmoset brain based on multi-modal MRI. NeuroImage, 169, 106–116. 10.1016/j.neuroimage.2017.12.004 PMC5856608 29208569

[IMAG.a.1419-b50] Loh, K. K., Petrides, M., Hopkins, W. D., Procyk, E., & Amiez, C. (2017). Cognitive control of vocalizations in the primate ventrolateral-dorsomedial frontal (VLF-DMF) brain network. Neuroscience and Biobehavioral Reviews, 82, 32–44. 10.1016/j.neubiorev.2016.12.001 27923733

[IMAG.a.1419-b51] Loh, K. K., Procyk, E., Neveu, R., Lamberton, F., Hopkins, W. D., Petrides, M., & Amiez, C. (2020). Cognitive control of orofacial motor and vocal responses in the ventrolateral and dorsomedial human frontal cortex. Proceedings of the National Academy of Sciences of the United States of America, 117(9), 4994–5005. 10.1073/pnas.1916459117/-/DCSupplemental PMC7060705 32060124

[IMAG.a.1419-b52] Long, M. A., Katlowitz, K. A., Svirsky, M. A., Clary, R. C., Byun, T. M. A., Majaj, N., Oya, H., Howard, M. A., & Greenlee, J. D. W. (2016). Functional segregation of cortical regions underlying speech timing and articulation. Neuron, 89(6), 1187–1193. 10.1016/j.neuron.2016.01.032 PMC4833207 26924439

[IMAG.a.1419-b53] MacNeilage, P. F. (1998). The frame/content theory of evolution of speech production. Behavioral and Brain Sciences, 21(4), 499–546. 10.1017/S0140525X98001265 10097020

[IMAG.a.1419-b54] Marcus, D. S., Harwell, J., Olsen, T., Hodge, M., Glasser, M. F., Prior, F., Jenkinson, M., Laumann, T., Curtiss, S. W., & Van Essen, D. C. (2011). Informatics and data mining tools and strategies for the human connectome project. Frontiers in Neuroinformatics, 5, 4. 10.3389/fninf.2011.00004 PMC3127103 21743807

[IMAG.a.1419-b55] Mathis, A., Mamidanna, P., Cury, K. M., Abe, T., Murthy, V. N., Mathis, M. W., & Bethge, M. (2018). DeepLabCut: Markerless pose estimation of user-defined body parts with deep learning. Nature Neuroscience, 21(9), 1281–1289. 10.1038/s41593-018-0209-y 30127430

[IMAG.a.1419-b56] Matsuzaki, M., & Ebina, T. (2020). Common marmoset as a model primate for study of the motor control system. Current Opinion in Neurobiology, 64, 103–110. 10.1016/j.conb.2020.02.013 32389894

[IMAG.a.1419-b57] Miller, C. T., Dimauro, A., Pistorio, A., Hendry, S., & Wang, X. (2010). Vocalization induced cFos expression in marmoset cortex. Frontiers in Integrative Neuroscience, 4, 128. 10.3389/fnint.2010.00128 PMC3004388 21179582

[IMAG.a.1419-b58] Miller, C. T., Freiwald, W. A., Leopold, D. A., Mitchell, J. F., Silva, A. C., & Wang, X. (2016). Marmosets: A neuroscientific model of human social behavior. Neuron, 90(2), 219–233. 10.1016/j.neuron.2016.03.018 PMC4840471 27100195

[IMAG.a.1419-b59] Miller, C. T., Thomas, A. W., Nummela, S. U., & De La Mothe, L. A. (2015). Responses of primate frontal cortex neurons during natural vocal communication. Journal of Neurophysiology, 114(2), 1158–1171. 10.1152/jn.01003.2014.-The PMC4725106 26084912

[IMAG.a.1419-b60] Miller, E. K., & Cohen, J. D. (2001). An integrative theory of prefrontal cortex function. Annual Review of Neuroscience, 24(1), 167–202. 10.1146/annurev.neuro.24.1.167 11283309

[IMAG.a.1419-b61] Montardy, Q., Kwan, W. C., Mundinano, I. C., Fox, D. M., Wang, L., Gross, C. T., & Bourne, J. A. (2021). Mapping the neural circuitry of predator fear in the nonhuman primate. Brain Structure and Function, 226(1), 195–205. 10.1007/s00429-020-02176-6 PMC7817595 33263778

[IMAG.a.1419-b62] Moore, J. D., Kleinfeld, D., & Wang, F. (2014). How the brainstem controls orofacial behaviors comprised of rhythmic actions. Trends in Neurosciences, 37(7), 370–380. 10.1016/j.tins.2014.05.001 PMC4100695 24890196

[IMAG.a.1419-b63] Morecraft, R. J., Louie, J. L., Herrick, J. L., & Stilwell-Morecraft, K. S. (2001). Cortical innervation of the facial nucleus in the non-human primate: A new interpretation of the effects of stroke and related subtotal brain trauma on the muscles of facial expression. Brain, 124(1), 176–208. 10.1093/brain/124.1.176 11133797

[IMAG.a.1419-b64] Morrill, R. J., Paukner, A., Ferrari, P. F., & Ghazanfar, A. A. (2012). Monkey lipsmacking develops like the human speech rhythm. Developmental Science, 15(4), 557–568. 10.1111/j.1467-7687.2012.01149.x PMC3383808 22709404

[IMAG.a.1419-b65] Moussavi, A., Mietsch, M., Drummer, C., Behr, R., Mylius, J., & Boretius, S. (2020). Cardiac MRI in common marmosets revealing age-dependency of cardiac function. Scientific Reports, 10(1), 10221. 10.1038/s41598-020-67157-5 PMC7311402 32576909

[IMAG.a.1419-b66] Müller-Preuss, P., & Jürgens, U. (1976). Projections from the ‘cingular’ vocalization area in the squirrel monkey. Brain Research, 103, 29–43. 10.1016/0006-8993(76)90684-3 56207

[IMAG.a.1419-b67] Müri, R. M. (2016). Cortical control of facial expression. Journal of Comparative Neurology, 524(8), 1578–1585. 10.1002/cne.23908 26418049

[IMAG.a.1419-b68] Nath, T., Mathis, A., Chen, A. C., Patel, A., Bethge, M., & Mathis, M. W. (2019). Using DeepLabCut for 3D markerless pose estimation across species and behaviors. Nature Protocols, 14(7), 2152–2176. 10.1038/s41596-019-0176-0 31227823

[IMAG.a.1419-b69] Paus, J. T., Perry, D. W., Zatorre, R. J., Worsley, K. J., & Evans, A. C. (1996). Modulation of cerebral blood flow in the human auditory cortex during speech: Role of motor-to-sensory discharges. European Journal of Neuroscience, 8, 2236–2246. 10.1111/j.1460-9568.1996.tb01187.x 8950088

[IMAG.a.1419-b70] Paxinos, G., Watson, C., Petrides, M., Rosa, M., & Tokuno, H. (2012). The marmoset brain in stereotaxic coordinates. Academic Press. https://shop.elsevier.com/books/the-marmoset-brain-in-stereotaxic-coordinates/paxinos/978-0-12-415818-4

[IMAG.a.1419-b71] Petrides, M., Cadoret, G., & Mackey, S. (2005). Orofacial somatomotor responses in the macaque monkey homologue of Broca’s area. Nature, 435(7046), 1235–1238. 10.1038/nature03628 15988526

[IMAG.a.1419-b72] Risueno-Segovia, C., & Hage, S. R. (2020). Theta synchronization of phonatory and articulatory systems in marmoset monkey vocal production. Current Biology, 30(21), 4276.e3–4283.e3. 10.1016/j.cub.2020.08.019 32888481

[IMAG.a.1419-b73] Rizzolatti, G., & Rozzi, S. (2018). The mirror mechanism in the parietal lobe. In Handbook of Clinical Neurology (Vol. 151, pp. 555–573). Elsevier B.V. 10.1016/B978-0-444-63622-5.00028-0 29519480

[IMAG.a.1419-b74] Robinson, B. W. (1967). Vocalization evoked from forebrain in Macaca Mulatta. Physiology and Behavior, 2(4), 345–354. 10.1016/0031-9384(67)90050-9

[IMAG.a.1419-b75] Roy, S., Zhao, L., & Wang, X. (2016). Distinct neural activities in premotor cortex during natural vocal behaviors in a new world primate, the common marmoset (*Callithrix jacchus*). Journal of Neuroscience, 36(48), 12169–12179. 10.1523/JNEUROSCI.1646-16.2016 PMC5148218 27903726

[IMAG.a.1419-b76] Rozzi, S., Ferrari, P. F., Bonini, L., Rizzolatti, G., & Fogassi, L. (2008). Functional organization of inferior parietal lobule convexity in the macaque monkey: Electrophysiological characterization of motor, sensory and mirror responses and their correlation with cytoarchitectonic areas. European Journal of Neuroscience, 28(8), 1569–1588. 10.1111/j.1460-9568.2008.06395.x 18691325

[IMAG.a.1419-b77] Sahara, Y., Kobayashi, T., Toya, H., & Suzuki, T. (2012). Applications of functional magnetic resonance imaging for analysis of oral functions. Journal of Oral Biosciences, 54(2), 101–106. 10.1016/j.job.2012.01.008

[IMAG.a.1419-b78] Sawczuk, A., & Mosier, K. (2001). Neural control of tongue movement with respect to respiration and swallowing. Critical Reviews in Oral Biology & Medicine, 12(1), 18–37. 10.1177/10454411010120010101 11349959

[IMAG.a.1419-b79] Schaeffer, D. J., Klassen, L. M., Hori, Y., Tian, X., Szczupak, D., Yen, C. C. C., Cléry, J. C., Gilbert, K. M., Gati, J. S., Menon, R. S., Liu, C. R., Everling, S., & Silva, A. C. (2022). An open access resource for functional brain connectivity from fully awake marmosets. NeuroImage, 252, 119030. 10.1016/j.neuroimage.2022.119030 PMC9048130 35217206

[IMAG.a.1419-b80] Schneider, D. M., Nelson, A., & Mooney, R. (2014). A synaptic and circuit basis for corollary discharge in the auditory cortex. Nature, 513(7517), 189–194. 10.1038/nature13724 PMC4248668 25162524

[IMAG.a.1419-b81] Schrago, C. G., & Russo, C. A. M. (2003). Timing the origin of New World monkeys. Molecular Biology and Evolution, 20(10), 1620–1625. 10.1093/molbev/msg172 12832653

[IMAG.a.1419-b82] Sessle, B. J., Adachi, K., Avivi-Arber, L., Lee, J., Nishiura, H., Yao, D., & Yoshino, K. (2007). Neuroplasticity of face primary motor cortex control of orofacial movements. Archives of Oral Biology, 52(4), 334–337. 10.1016/j.archoralbio.2006.11.002 17174267

[IMAG.a.1419-b83] Sessle, B. J., Yao, D., Nishiura, H., Yoshino, K., Lee, J.-C., Martin, R. E., & Murray, G. M. (2005). Properties and plasticity of the primate somatosensory and motor cortex related to orofacial sensorimotor function. Clinical and Experimental Pharmacology and Physiology, 32(1–2), 109–114. 10.1111/j.1440-1681.2005.04137.x 15730444

[IMAG.a.1419-b84] Sherwood, C. C., Hof, P. R., Holloway, R. L., Semendeferi, K., Gannon, P. J., Frahm, H. D., & Zilles, K. (2005). Evolution of the brainstem orofacial motor system in primates: A comparative study of trigeminal, facial, and hypoglossal nuclei. Journal of Human Evolution, 48(1), 45–84. 10.1016/j.jhevol.2004.10.003 15656936

[IMAG.a.1419-b85] Siebert, S., & Jürgens, U. (2003). Vocalization after periaqueductal grey inactivation with the GABA agonist muscimol in the squirrel monkey. Neuroscience Letters, 340(2), 111–114. 10.1016/S0304-3940(03)00071-5 12668249

[IMAG.a.1419-b86] Simões, C. S., Vianney, P. V. R., de Moura, M. M., Freire, M. A. M., Mello, L. E., Sameshima, K., Araújo, J. F., Nicolelis, M. A. L., Mello, C. V., & Ribeiro, S. (2010). Activation of frontal neocortical areas by vocal production in marmosets. Frontiers in Integrative Neuroscience, 4, 123. 10.3389/fnint.2010.00123 PMC2955454 20953246

[IMAG.a.1419-b87] Simonyan, K., & Jürgens, U. (2003). Efferent subcortical projections of the laryngeal motorcortex in the rhesus monkey. Brain Research, 974(1–2), 43–59. 10.1016/S0006-8993(03)02548-4 12742623

[IMAG.a.1419-b88] Smith, S. M., Jenkinson, M., Woolrich, M. W., Beckmann, C. F., Behrens, T. E. J., Johansen-Berg, H., Bannister, P. R., De Luca, M., Drobnjak, I., Flitney, D. E., Niazy, R. K., Saunders, J., Vickers, J., Zhang, Y., De Stefano, N., Brady, J. M., & Matthews, P. M. (2004). Advances in functional and structural MR image analysis and implementation as FSL. NeuroImage, 23, S208–S219. 10.1016/j.neuroimage.2004.07.051 15501092

[IMAG.a.1419-b89] Smith, W. K. (1945). The functional significance of the rostral cingular cortex as revealed by its responses to electrical excitation. Journal of Neurophysiology, 8(4), 241–255. 10.1152/jn.1945.8.4.241

[IMAG.a.1419-b90] Sörös, P., Sokoloff, L. G., Bose, A., McIntosh, A. R., Graham, S. J., & Stuss, D. T. (2006). Clustered functional MRI of overt speech production. NeuroImage, 32(1), 376–387. 10.1016/j.neuroimage.2006.02.046 16631384

[IMAG.a.1419-b91] Stevenson, M. F., & Poole, T. B. (1976). An ethogram of the common marmoset (*Calithrix jacchus*): General behavioural repertoire. Animal Behaviour, 24(2), 428–451. 10.1016/s0003-3472(76)80053-x 820223

[IMAG.a.1419-b92] Travers, J., Dinardo, L. A., & Karimnamazi, H. (1997). Motor and premotor mechanisms of licking. Neuroscience and Biobehavioral Reviews, 21(5), 631–647. 10.1016/s0149-7634(96)00045-0 9353796

[IMAG.a.1419-b93] Tsunada, J., & Eliades, S. J. (2025). Frontal-auditory cortical interactions and sensory prediction during vocal production in marmoset monkeys. Current Biology, 35(10), 2307.e3–2322.e3. 10.1016/j.cub.2025.03.077 PMC13286032 40250436

[IMAG.a.1419-b94] Vázquez, Y., Ianni, G. R., Rassi, E., Rouse, A. G., Schieber, M. H., Yazdani, F., Prut, Y., & Freiwald, W. A. (2025). Neural synchrony links sensorimotor cortices in a network for facial motor control. Proceedings of the National Academy of Sciences of the United States of America, 122(52), e2512604122. 10.1073/pnas.2512604122 PMC12772211 41433067

[IMAG.a.1419-b95] Von Cramon, D., & Jürgens, U. (1983). The anterior cingulate cortex and the phonatory control in monkey and man. Neuroscience & Biobehavioral Reviews, 7(3), 423–425. 10.1016/0149-7634(83)90049-0 6422356

[IMAG.a.1419-b96] West, R. A., & Larson, C. R. (1995). Neurons of the anterior mesial cortex related to faciovocal activity in the awake monkey. Journal of Neurophysiology, 74(5), 1856–1869. 10.1152/jn.1995.74.5.1856 8592179

[IMAG.a.1419-b97] Zanini, A., Dureux, A., Jafari, A., Gilbert, K. M., Zeman, P., Bellyou, M., Li, A., Tuin, C. Vander, & Everling, S. (2023). In vivo functional brain mapping using ultra-high-field fMRI in awake common marmosets. STAR Protocols, 4(4), 102586. 10.1016/j.xpro.2023.102586 PMC10520676 37738120

[IMAG.a.1419-b98] Zhang, H., Zhu, Z., Ma, W. X., Kong, L. X., Yuan, P. C., Bu, L. F., Han, J., Huang, Z. L., & Wang, Y. Q. (2024). The contribution of periaqueductal gray in the regulation of physiological and pathological behaviors. Frontiers in Neuroscience, 18, 1380171. 10.3389/fnins.2024.1380171 PMC11034386 38650618

[IMAG.a.1419-b99] Zhao, L., & Wang, X. (2023). Frontal cortex activity during the production of diverse social communication calls in marmoset monkeys. Nature Communications, 14(1), 6634. 10.1038/s41467-023-42052-5 PMC10587070 37857618

