# Supplementary material for: Oromotor functional networks in the marmoset monkey
Source: Imaging Neurosci (Camb). 2026 Oct 26;4:IMAG.a.1419. doi: 10.1162/IMAG.a.1419 (PMC13710931; doi:10.1162/IMAG.a.1419)
Supplement: Supplementary file 1 [file IMAG.a.1419_supp.pdf]

Supplementary Figures

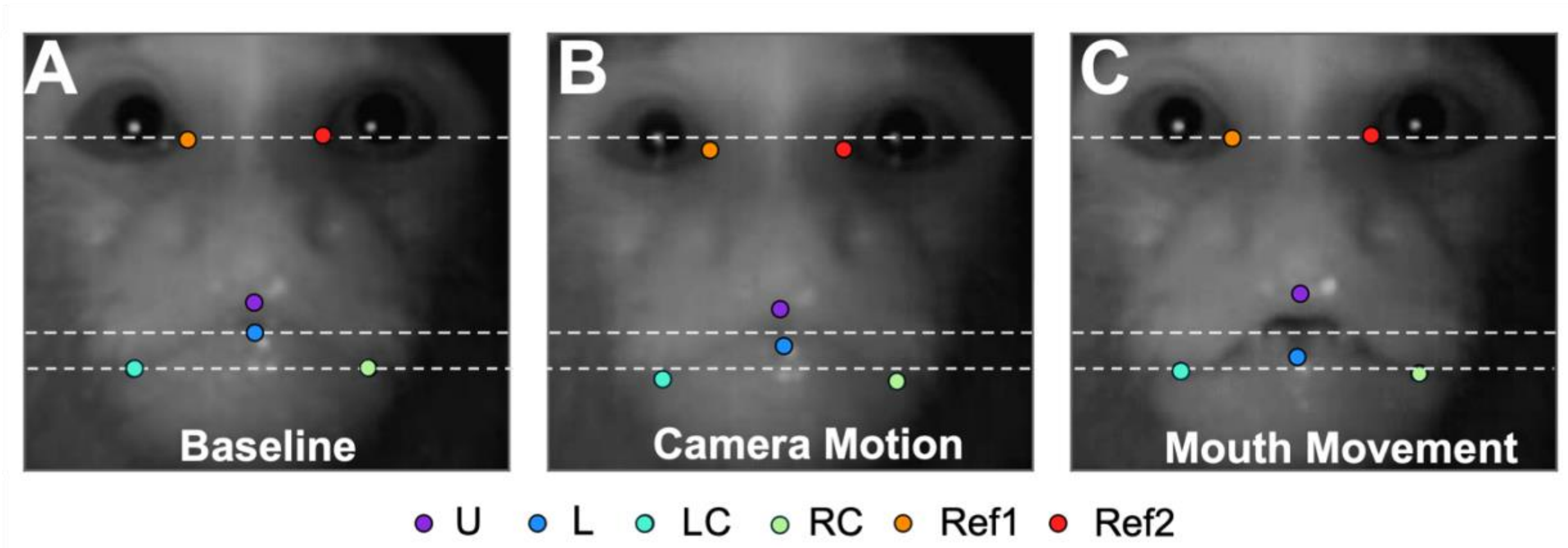

Figure S1. Camera motion can mimic a real articulator movement. Video frames from a representative run, shown at identical image coordinates, with the six facial landmarks displayed (colors as in Fig. 1C). The dashed horizontal lines mark the mean positions across the run of the eye-corner references (top), the lower lip (middle), and the mouth corners (bottom). (A) Baseline: the mouth is closed, and the camera is stationary; reference, lower lip and mouth corner landmarks lie close to the run-mean lines. (B) Baseline frame with camera motion: the mouth remains closed, but the entire set of facial landmarks is displaced relative to the dashed lines. The accompanying displacements of the lower lip and mouth corners are therefore artifactual, yet in the uncorrected trace they might exceed the detection threshold for this specific run and would then be counted as an oromotor event. **(C)** Actual mouth movement: the animal opens its mouth while the camera is stationary in this frame; Ref1 and Ref2 have remained close to the dashed line and only the articulator landmarks are displaced. U (upper lip midpoint), L (lower lip midpoint), LC (left mouth corner), RC (right mouth corner), Ref1 and Ref2 (inner tips of the left and right eye).

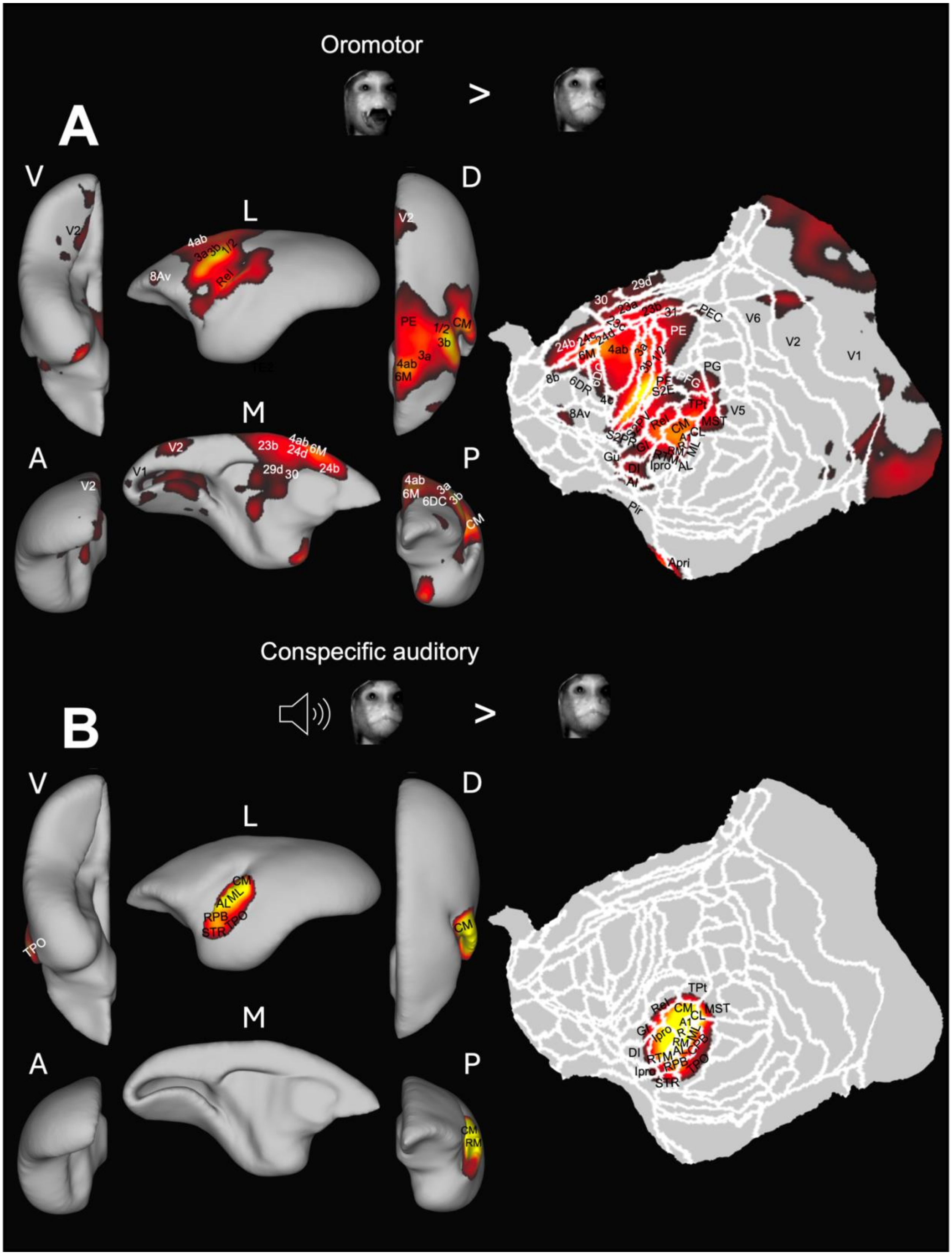

Oromotor
>
A
V
L
D
M
A
P
V2
4ab
3a
3b
1/2
8Av
Rel
PE
CM
4ab
6M
23b
24d
24b
29d
30
V1
6DC
PEC
V6
V1
PG
S2E
TPt
MST
V5
8b
6DR
4c
Gu
DI
AL
ML
CL
A1
Ipro
Pir
Apri
Conspecific auditory
>
B
V
L
D
M
A
P
TPO
CM
AL
ML
RPB
STR
RM
TPt
MST
Ipro
RTM

1

Figure S2. Left hemisphere cortical networks (Oromotor vs. baseline and Auditory vs. baseline). (A) Group activation maps for *oromotor* > *baseline*, displayed on left hemisphere cortical surfaces (views: D, V, M, L, A, P) and flatmap (analogous to Fig. 2A for the right hemisphere). (B) *Conspecific auditory* > *baseline* for left hemisphere (analogous to Fig. 2B). The left hemisphere shows patterns similar to the right, with bilateral involvement of motor, cingulate, and parietal areas in the oromotor condition, and bilateral auditory cortex activation in the auditory condition.

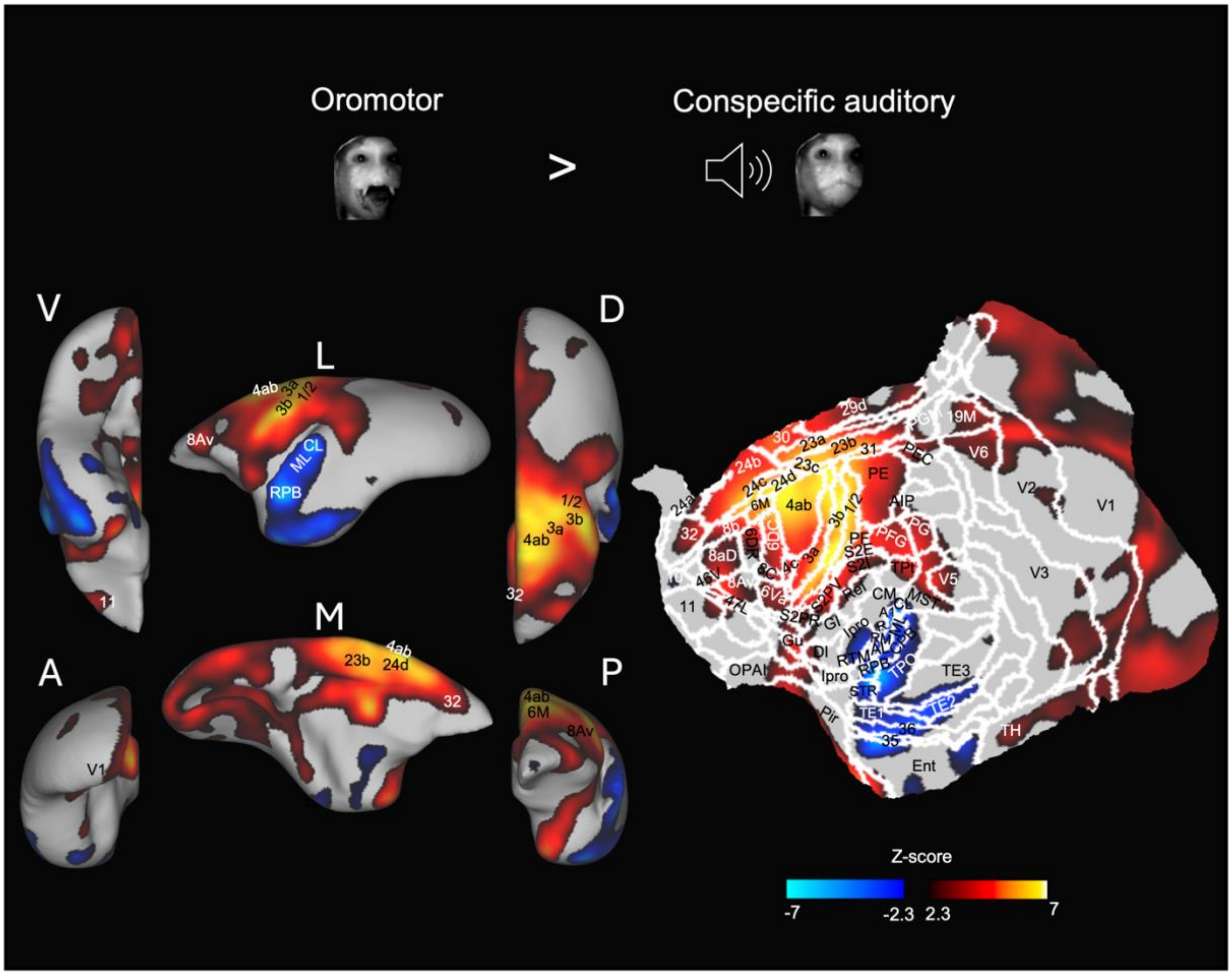

Figure S3. Left hemisphere cortical networks (Oromotor vs. Auditory). Group contrast maps for *oromotor movements* > *conspecific auditory* on left hemisphere surfaces (views: D, V, M, L, A, P) and flatmap (analogous to Fig. 5 for right hemisphere). Warm colors indicate regions with greater activation during oromotor movements; cool colors indicate the reverse. The left hemisphere exhibits the same trends as described for the right: greater engagement of motor, premotor, and ACC regions for oromotor behavior, versus greater auditory cortex activation for call listening.

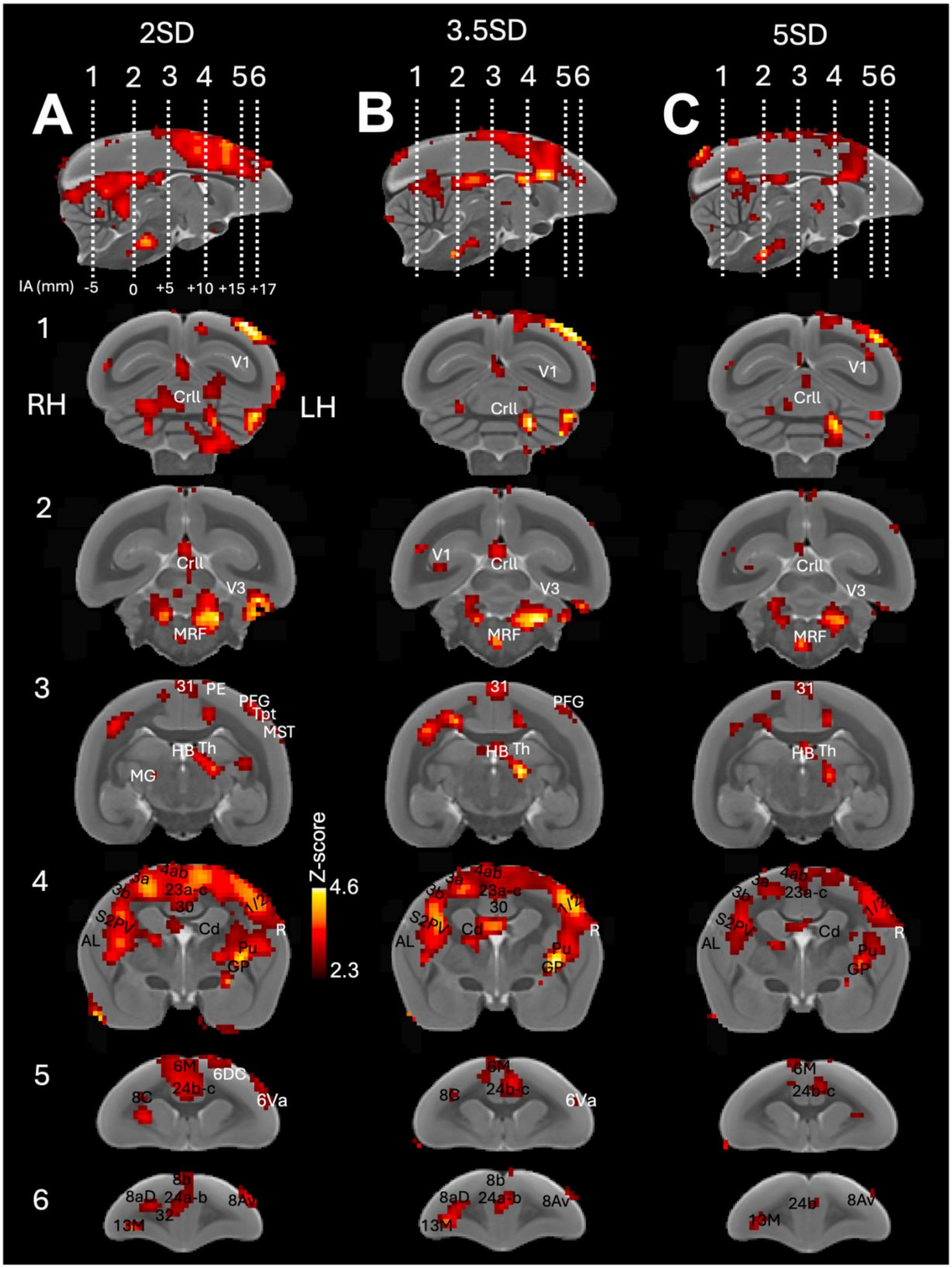
2SD
3.5SD
5SD
1 2 3 4 56
A
B
C
IA (mm) -5 0 +5 +10 +15 +17
RH
LH
V1
Crll
MRF
V3
31
PE
PFG
Tpt
MST
HB
Th
MG
Z-score
4.6
2.3
4ab
3a
3b
23a-c
30
S2PV
AL
Cd
Pu
GP
R
AI2
6M
6DC
24b-c
8C
6Va
8b
8aD
24a-b
32
13M
8Av
24b

***Figure S4. Robustness of the oromotor network to event-detection threshold*.** Group activation maps for the *oromotor* > *baseline* generated using events detected at fixed thresholds of (A) 2 SD, (B) 3.5 SD, and (C) 5 SD from the camera-motion-corrected articulator traces, with false candidates removed by manual review. Colors denote *Z*-scores (2.3–4.6), and maps are displayed on sagittal view (first row) and coronal slices at different interaural (IA) levels (bottom rows). To maintain the number of runs and monkeys fixed across different thresholds, the analysis was restricted to 43 runs from 9 monkeys in which events were detected at all three thresholds, resulting in a total number of 1,332 events at 2 SD, 870 at 3.5 SD, and 648 at 5 SD. The 5 SD events are a subset of the 3.5 SD events, which are a subset of the 2 SD events. The sensorimotor, cingulate, subcortical, and brainstem regions of the oromotor network are activated across all three thresholds, while the spatial extent of significant activation decreases progressively at higher thresholds. Crll (cerebellum), Cd (caudate), MRF (medullary reticular formation), Th (thalamus), Pu (putamen), GP (globus pallidus), MG (medial geniculate nucleus), HB (habenular nuclei), LH (left hemisphere), RH (right hemisphere).
